# Application and evaluation of CRACMM v1.0 mechanism in PM_2.5_ simulation over China

**DOI:** 10.5194/gmd-19-2531-2026

**Published:** 2026-03-31

**Authors:** Qingfang Su, Yifei Chen, Yangjun Wang, David C. Wong, Havala O. T. Pye, Ling Huang, Golam Sarwar, Benjamin Murphy, Bryan Place, Li Li

**Affiliations:** 1 Key Laboratory of Organic Compound Pollution Control Engineering (MOE), School of Environmental and Chemical Engineering, Shanghai University, Shanghai, 200444, China; 2 Office of Research and Development, U.S. Environmental Protection Agency, Research Triangle Park, North Carolina, USA; 3 Department of Earth and Atmospheric Sciences, University of Houston, Houston, USA; 4 Oak Ridge Institute for Science and Engineering (ORISE), Office of Research and Development, U.S. Environmental Protection Agency, Research Triangle Park, North Carolina, USA

## Abstract

Chemical mechanisms are one of the major sources of bias in chemical transport model simulations, making their improvement a critical step towards enhancing model performance and supporting air quality management and research. In this study, a newly developed chemical mechanism, the Community Regional Atmospheric Chemistry Multiphase Mechanism (CRACMM), integrated into the Community Multiscale Air Quality (CMAQ) modeling system, was evaluated through comparison with two traditional chemical mechanisms, Carbon Bond 6 version r3 with aero7 treatment of SOA (CB6r3_ae7) and State Air Pollution Research Center version 07tc with extended isoprene chemistry and aero7i treatment of SOA (Saprc07tic_ae7i), for China. Sensitivity simulations related to precursor reactive organic carbon (ROC) emissions were conducted to investigate the key driving factors of PM_2.5_ formation. The results indicate that, when using the traditional primary organic aerosol (POA) inventory, the differences among the three chemical mechanisms are within 0–0.14 for the *R*, 0–10 μg m^−3^ for the MB, and within 10 % for the NMB values. However, when the full-volatility emission inventory is applied in January, CRACMM exhibits improved performance in the Pearl River Delta (PRD) region. The MB is reduced by 3.0–7.8 μg m^−3^. In addition, the NMB decreases by 17 %–23 %, and the root mean square error (RMSE) is reduced by 1–6 μg m^−3^ compared with simulations using the traditional POA inventory across the four months. CRACMM predicts higher PM_2.5_ concentrations during spring, summer and autumn, mainly due to enhanced secondary organic aerosol (SOA) formation driven by increased precursor emissions. Benzene–toluene–xylene (BTX) species and semi-volatile organic compound (SVOC) emissions significantly contributed to PM_2.5_ formation in CRACMM. The SOA from BTX emissions accounts for nearly 50 % of the PM_2.5_ changes, while intermediate-volatility organic compounds (IVOC) and SVOC emissions mainly affect PM_2.5_ concentrations through SOA formation. These results indicate that CRACMM, when using the full-volatility inventory, can effectively compensate for the underestimation of PM_2.5_ mass that may occur with traditional POA treatment, particularly in regions with high photochemical activity and abundant S/IVOC precursors.

## 1 Introduction

Exposure to airborne PM_2.5_ is associated with a variety of harmful health effects (Liu et al., 2024; Tsai et al., 2025; Kim et al., 2015) and was reported to cause 4.14 million deaths worldwide annually (95 % confidence interval: 3.45 to 4.80) (Murray et al., 2020), underscoring the urgent need for effective mitigation of PM_2.5_ pollution. Thus, a better understanding of the PM_2.5_ formation mechanism is essential for formulating effective air pollution control strategies.

Chemical transport models such as the Community Multiscale Air Quality (CMAQ) model (Byun and Schere, 2006) have been widely applied in China to investigate air quality issues, including seasonal PM_2.5_ and ozone distributions and their formation mechanisms. The CMAQ version 5.02 and 5.3.2, both versions are equipped with the gas-phase mechanism of State Air Pollution Research Center version99 (Saprc99) and Carbon Bond 6 version (CB6). These two chemical mechanisms were employed to simulate air quality over China during 2013–2019 (Mao et al., 2022). In that study, PM_2.5_ was examined in the North China Plain (NCP), Yangtze River Delta (YRD), and Pearl River Delta (PRD), while it was overestimated in the Chengyu Basin (CY) and Fen-Wei Plain (FWP) regions with NMB and NME values greatly exceeding the suggested criteria. In addition, the FWP and CY also showed lower *R* than the benchmark (*R* < 0.4) suggested by Emery et al during heavily polluted episode (Emery et al., 2017). During 2014–2017, the Comprehensive Air Quality Model with Extension (CAMx) (Yarwood et al., 2007) version 6.2, version 7.1, CMAQv5.0.2, and CMAQv5.3.2 models performed quite well in PM_2.5_ mean performance. The bias and error terms of the four models were resemblant small: −0.29, −0.07, −0.04, and −0.11 for NMB and 0.51, 0.48, 0.53, and 0.52 for NME, 0.58, 0.55, 0.60, and 0.39, for *R* respectively (Meng et al., 2026), the CAMx6.2, CAMx7.1, and CMAQv5.0.2 simulations covered the entire country, whereas CMAQv5.3.2 was evaluated over eastern China. (Meng et al., 2026). According to Kang et al.’s research, the model shows good performance for PM_2.5_ in most areas, except for the PRD region, where the mean fractional biases (MFB) for PM_2.5_ using Saprc series mechanisms are slightly outside the recommended range (Boylan and Russell, 2006). According to Huang’s research, evaluation results of PM_2.5_ simulation by SOAP3 in CAMx in BTH, YRD, FWP, SCB and PRD areas, the *R* values are 0.31, 0.45, 0.38, 0.48, and 0.15 in July, respectively. In November, the *R* values are also low for PRD and FWP, 0.30 and 0.48, respectively (Huang et al., 2024).

Existing studies indicate that different chemical mechanisms vary significantly in terms of the number of species, reaction complexity, and the coverage of chemical pathways, could lead to noticeable differences in regional air quality model performance. With this realization, we further examined the limitations of the commonly used chemical mechanisms (CB, Saprc and Regional Atmospheric Chemical Mechanism, RACM). In CB mechanism, model species represent the concentrations of constituent groups regardless of the molecule to which they are attached. Initially, this approach conserved carbon atoms, required relatively few species, and both led to lower computational costs. However, as the mechanism evolved, its grouping increasingly resembled aggregated molecule schemes, since both molecular structure and total molecular weight significantly influence atmospheric chemistry (Yarwood et al., 2005). The disadvantage of CB mechanism is that chemical expression of free radicals is insufficient (Kang et al., 2016; Sarwar et al., 2008). Comparative analysis of different CB6 mechanism variants shows that differences in reaction pathways can lead to significant deviations in model predictions of ozone, NO_*x*_, and formaldehyde (Cao et al., 2021).

The Saprc series (Saprc −90, Saprc −99, Saprc −07) aggregate VOCs by molecule or functional group, representing roughly 400 categories. Condensed versions are widely used in urban and regional air quality models, but simplifications can limit the accuracy of organic chemistry representation (Stockwell et al., 2012). Comparative analyses of different Saprc variants reveal discrepancies in predicting ozone, radical species, and oxidative products, indicating uncertainties arising from mechanism and emission inventories (Kang et al., 2025). For RACM, it was developed for broader applications, where NO_*x*_ is lower and slower-reacting organics are more important. RACM version 2 includes 118 species and 356 reactions. Even though this mechanism is computationally efficient, it may inadequately capture detailed organic chemistry and secondary organic aerosol (SOA) formation, resulting in potential uncertainties in air quality predictions (Stockwell et al., 2012).

In air quality models, gas-phase chemical mechanisms are typically coupled with aerosol modules to simulate interactions between the gas and particulate phases. Aerosol Module 7 (aero7) is the latest aerosol representation within the CMAQ model (Byun and Schere, 2006), developed by the US Environmental Protection Agency (EPA). Aero7 improves consistency in representing SOA formation pathways between the CB- and SAPRC-based chemical mechanisms. It also updates monoterpene SOA yields from photooxidation, adds uptake of water onto hydrophilic organics, and includes consumption of inorganic sulfates ${(\text{SO}}_{4}^{2-})$ when isoprene epoxydiol (IEPOX) organosulfates are formed (Pye et al., 2013). Furthermore, it enhances computational efficiency by using a volatility basis set (VBS) to parameterize SOA yields rather than using the Odum 2-product fit (Zhang et al., 2021a).

Most of the above chemical mechanisms and SOA treatment exhibit substantial limitations in simulating SOA formation (Wang et al., 2019; Zhang et al., 2021b, 2022, 2023; Huang et al., 2024). First, Semi-Volatile Organic Compounds (SVOC) and Intermediate-Volatility Organic Compounds (IVOC) species are not explicitly represented, leading to an underestimation of SOA contribution from those precursors. Even in 2D-VBS mechanisms, which includes S/IVOC species, the gas-to-particle conversion of oxidative products are mainly characterized using empirical parameters (e.g., yields and volatility distributions). Moreover, the 2D-VBS framework does not explicitly track individual chemical reactions; instead, it relies on parameterizations derived from environmental chamber experiments or model calibration data, making it difficult to resolve specific chemical pathways of SOA formation (Chang et al., 2022). Second, the use of fixed-yield empirical parameterization limits the representation of multigenerational oxidative processes and gas-particle partitioning dynamics (Chang et al., 2022). Third, POA is commonly assumed to be non-volatile and non-reactive, thereby neglecting its potential for re-evaporation and subsequent oxidation to form SOA. These simplifications overly idealize SOA formation processes missing various key reactions and resulted in an inaccurate representation of the complex aging of organic aerosols in the atmosphere (Huang et al., 2024).

The Community Regional Atmospheric Chemistry Multiphase Mechanism (CRACMM) is the latest chemical mechanism developed under the leadership of scientists at the US EPA. CRACMM is built upon version 2 of RACM (Goliff et al., 2013) and incorporates state-of-the-science developments, including autoxidation, aromatic chemistry, oxygenated hydrocarbons, organic nitrates, and halogen chemistry. These advances enhance the representation of atmospheric chemical transformations, enabling more realistic simulations of key air pollutants such as O_3_, PM_2.5_, and various hazardous species, e.g., formaldehyde (Skipper et al., 2024). In addition, CRACMM integrates a full-volatility organic framework and explicitly accounts for multigenerational oxidative processes, thereby improving the physicochemical representation of secondary organic aerosol (SOA) formation and providing a more comprehensive description of SOA evolution (Ng et al., 2008).

CRACMM also provides detailed species mapping methodology between emission inventory and chemical mechanism (Pye et al., 2023a), to ensure carbon conservation when tracking the transformation of carbon from emission sources to products (Ng et al., 2008). CB6r3_ae7 and Saprc07tic_ae7i do not consider certain SOA precursors such as L/S/IVOC (Chang et al., 2022), and CRACMM explicitly accounts for SOA precursors beyond traditional non-oxygenated volatile hydrocarbons, including phenolic compounds, furans, and other oxygenated organic species (Pye et al., 2023a). This allows CRACMM describing and simulating SOA in more precise and accurate manner. No doubt CRACMM a good chemical mechanism candidate to address the above listed shortcomings. CRACMMv1.0 was previously applied in summer simulations over the northeastern United States, as reported by Place et al. (2023), and the result showed ozone simulated values were better than RACM2_ae6 chemical mechanism in terms of comparing with observation: the average bias of RACM2_ae6 is +4.2 ppb, average bias of CRACMMv1.0 +2.1 ppb. A few studies have been conducted using CRACMM but they all focused on US CONUS domain (Place et al., 2023). CRACMM has not yet been applied on China domain so thorough evaluation is a must.

For computational cost considerations, the number of species and reactions directly affects model runtime. Mechanisms with larger numbers of species and reactions, such as Saprc07tic_ae7i, which contains the largest number of reactions and species among the standard CMAQ mechanisms, is the most computationally expensive mechanism compared to simpler mechanisms like CB6r3_ae7. In contrast, CB has fewer reactions and species. The primary difference between CB6r3_ae7 and Saprc07tic_ae7i, and CRACMM lies in the chemistry module. Our tests indicate that CRACMM requires approximately 30 %–40 % more computational time than CB6r3_ae7, but 20 %–30 % less than Saprc.

With advances in high-performance computing, CRACMM could also be applied on a global scale. Particularly, the Model for Prediction Across Scales (MPAS) global meteorological model has recently been successfully coupled with CMAQ, demonstrating the application of CRACMM in the global MPAS-CMAQ coupled model framework (Wong et al., 2024). Also, CRACMM is not restricted to CMAQ. The design of CRACMM follows a modular framework for gas-phase chemistry and SOA formation, which does not rely on model-specific assumptions. Therefore, in principle, CRACMM could be implemented in other regional models, although additional effort would be required to adapt emission mapping and aerosol–chemistry coupling.

In this study, PM_2.5_ predictions from the CRACMM mechanism were evaluated with surface observations comprehensively, covering different seasons and regions. Results derived by CRACMM are compared with two well-established chemical mechanisms, Saprc07tic_ae7i and CB6r3_ae7. The differences in PM_2.5_ and SOA drivers between CRACMM and the two existing mechanisms are further explored. The results of this study provide a solid foundation for the further application of CRACMM in understanding and regulating air pollution in China and globally.

## 2 Methodology

### 2.1 Model configuration

**Figure 1.**
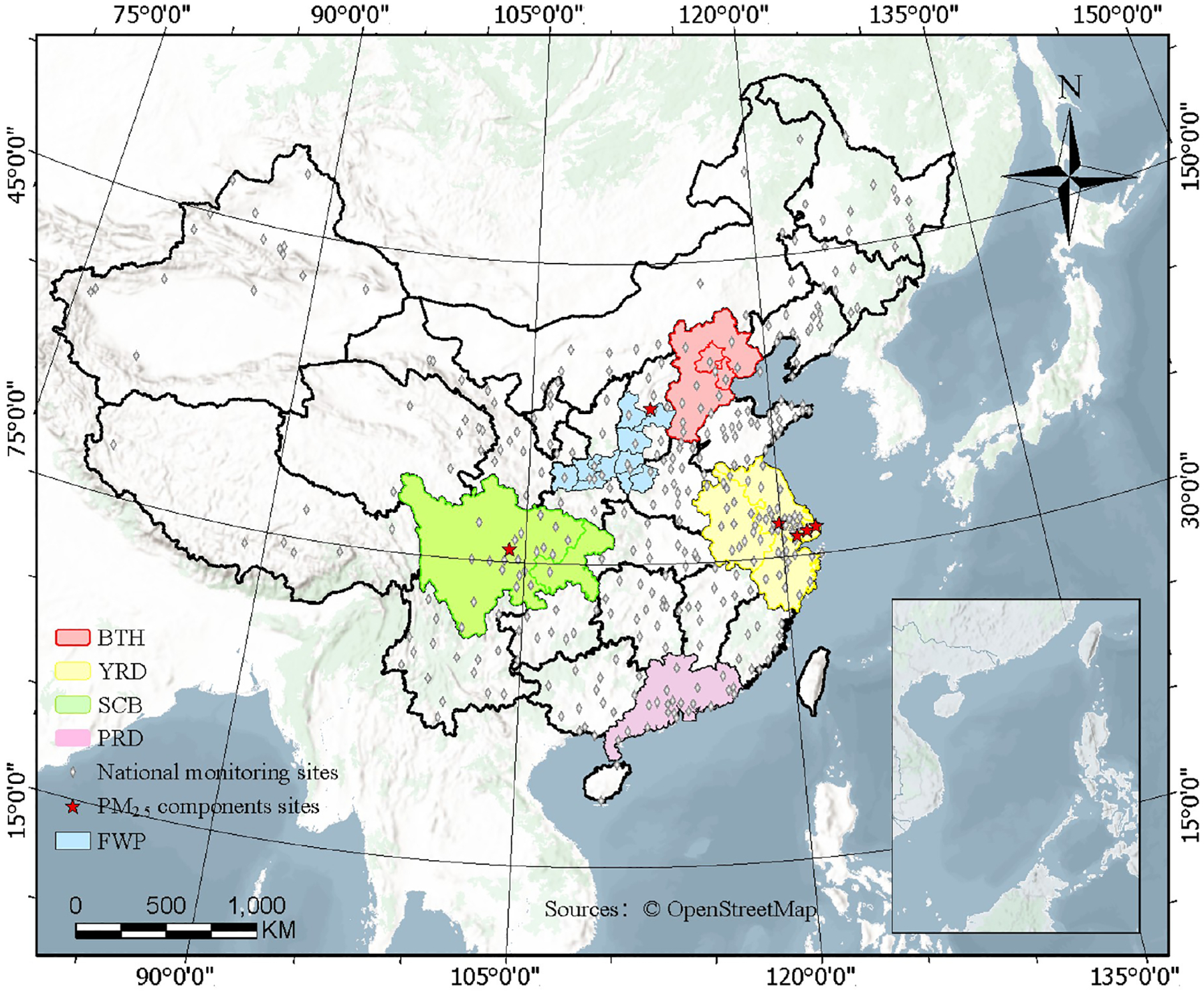
Model domain with five key city clusters (outlined in color), locations of national monitoring sites (grey diamonds), and six PM_2.5_ chemical components observation sites (red stars).

Model simulations were conducted using CMAQ v5.4 with a horizontal resolution of 36 km × 36 km, covering mainland China (Fig. 1). This domain includes five key city clusters with notable air pollution levels (Deng et al., 2022; Huang et al., 2024): Beijing-Tianjin-Hebei (BTH), Yangtze River Delta (YRD), Pearl River Delta (PRD), Fen-Wei Plain (FWP), and Sichuan Basin (SCB). Simulations were carried out for the months of January, April, July, and October 2021, representing winter, spring, summer, and autumn, respectively. The model includes 34 vertical layers, with the first layer located approximately 35 m a.g.l. Each simulation was initialized with a 15 d spin-up period before the start of each month. In addition to CRACMM version 1.0, two other chemical mechanisms, CB6r3_ae7 and Saprc07tic_ae7i, were included for comparisons, the number of reactions and gas- and particle-phase species in three different chemical mechanisms used in CMAQ are shown in Fig. S1 in the Supplement.

Simple flowcharts illustrating the different species represented in each mechanism and their sources and sinks are shown in Fig. S2 for CRACMM and Fig. S3 for CB6r3_ae7. For Saprc07tic_ae7i,it has been described in detail in a previous study (Pye et al., 2015). All three mechanisms are available in CMAQv5.4, with the “m3dry” deposition scheme selected. The initial and boundary conditions for CRACMM and Saprc07tic_ae7i were mapped from the seasonal average hemispheric CMAQ output files distributed through the CMAS. CMAQ ready meteorological input files were created by the Meteorology-Chemistry Interface Processor (MCIP) (Otte and Pleim, 2010) version 5.4 processing through output files from an offline run of the Weather Research and Forecasting (WRF) model version 4.0 (Skamarock et al., 2019). WRF configuration was detailed in our previous studies (Huang et al., 2021a). The archived dataset, including the concentrations and model performance statistics of PM_2.5_ and its components, model configurations, and the locations of all observation sites, is available on Zenodo (Su and Chen, 2025).

### 2.2 Emissions

#### 2.2.1 Traditional Emissions Inventory

The 2019 anthropogenic Multi-resolution Emission Inventory for China (MEIC), developed by Tsinghua University, was utilized in this study (http://www.meicmodel.org, last access: 26 March 2026). Its spatial resolution is 0.25° × 0.25° and includes five sectors: power, industry, residential, transportation, and agriculture, and the provincial VOC emissions in 2019 from MEIC, categorized by sector for January, April, July, October are shown in Tables S1–S4. Biogenic emissions were estimated using the Model of Emissions of Gases and Aerosols from Nature version 3.2 (MEGANv3.2) (Guenther et al., 2012). Currently, MEIC supports VOC emission only for both CB6r3_ae7 and Saprc07tic_ae7i, but not the CRACMM mechanism. Therefore, anthropogenic VOC species were converted to CRACMM input species using a binary decision tree approach. This approach distinguishes between one-to-one and non-one-to-one mappings based on chemical species correspondence from Saprc07tic_ae7i and CB6r3_ae7 to CRACMM. The one-to-one mappings are further classified into explicit one-to-one (routine A) and lumped one-to-one (routine B), while the non-one-to-one mappings include many-to-one (routine C) and many-to-many (routine D) cases. In routine A, both Saprc07tic_ae7i and CB6r3_ae7 consist of a few species that can be mapped directly to CRACMM based on CAS number, e.g., HCHO (formaldehyde) is mapped to HCHO. In routine B, the mapping is based on lumped species categories or names. For example, OLE1 (alkenes other than ethene, with *k*OH < 7 × 10^4^ ppm^−1^ min^−1^) in Saprc07tic_ae7i is mapped to OLI (internal alkenes) in CRACMM, OLE2 (alkenes with *k*OH > 7 × 10^4^ ppm^−1^ min^−1^) is mapped to OLT (terminal alkenes), and ONIT is mapped to RNO3 based on the same name used for organic nitrates.

In routine C, new species could be added to the CRACMM mechanism, such as CSL (Cresols) and PHEN (Phenol and aromatic diols), which correspond to CRES (phenols and cresols) in the Saprc07tic_ae7i mechanism. In this case, we use the emission factor ratio to distribute the species. As MEIC does not provide species-level emission factors, data from the 2017 U.S. National Emission Inventory (NEI) (Pye et al., 2023b) were utilized, which contain over 3000 species with corresponding emission factors, source sectors, and CRACMM species mappings. The emission factors are averaged over all sources for different MEIC sectors (mobile sources, industrial sources, etc.). Another example is that both XYE (*p*-xylene and less reactive aromatics) and XYM (*m*-xylene and more reactive aromatics) are newly introduced species in the CRACMM mechanism, corresponding to XYL (Xylene and other aromatics) in the Saprc07tic_ae7i mechanism. According to the 2017 NEI, their ratio is 0.3 : 0.7. Therefore, 0.3 of XYL in Saprc07tic_ae7i is assigned to XYE in CRACMM, and 0.7 of XYL in Saprc07tic_ae7i is assigned to XYM in CRACMM.

Routine D is more complicated, but the mapping is still based on the emission factor ratio for proper mapping. For instance, in CRACMM, GLY represents both glyoxal and glycolaldehyde. To construct this species from Saprc07tic_ae7i, GLY (representing glyoxal only) and part of CCHO (glycolaldehyde and acetaldehyde) are mapped, such that GLY in CRACMM corresponds to GLY + CCHO × 0.25 in Saprc07tic_ae7i. Similarly, ACD (acetaldehyde) in CRACMM corresponds to CCHO × 0.75 in Saprc07tic_ae7i. For species lumped from multiple species, only those with larger emission factors are considered. Table S5 outlines the correspondence relationships for major species with substantial emissions. Since it is challenging to compare VOC emissions in different mechanisms due to the lumping rules, we only conducted an overall comparison of total emissions, as shown in Table S6.

#### 2.2.2 POA Emissions

Two POA inventories were employed in this study: a traditional POA emissions inventory and a full-volatility inventory. CRACMM, Saprc07tic_ae7i, and CB6r3_ae7 all use the same dataset for the traditional POA emissions inventory. This inventory applies a VBS profile based on Woody et al. (2016) and Robinson et al. (2007), treating POA as semi-volatile with $C_{i}^{*}$ values ranging from 10^−2^ to 10^3^ μg m^−3^. The detailed species of POA included in each mechanism are listed in Table S7.

In contrast, the full-volatility inventory distributes POA emissions across a wider range of volatility bins. Laboratory experiments have demonstrated that L/S/IVOC emissions, which are largely absent in the traditional POA inventory, contribute to SOA formation much more efficiently than VOCs, owing to their lower volatility. To capture these processes, the full-volatility inventory developed by Chang et al. (2022) was used. In this study, all species mapped from the full-volatility inventory (implemented using the two-dimensional VBS (2D-VBS) framework) to CRACMM are classified into two lumped categories: (1) an alkane-like ROC group, (2) 15 CRACMM mechanism species representing oxygenated S/IVOCs. Since all species exist in both the particle and gas phases, the same mapping rules are applied. The mapping rules for the gas phase are summarized in Table S8.

For eight new alkane-like ROC species with high OA formation potential spanning the L/S/IVOC range and are grouped by ${\text{log}}_{10}\left(C_{i}^{*}\right)$ into ROCN1ALK, ROCP0ALK, VROCP1ALK, ROCP2ALK, ROCP3ALK, ROCP4ALK, ROCP5ALK, and ROCP6ALK. They are mapped from CSM1O2C00P, CS00O2C00P, CS01O2C00P, CS02O2C00P, CS03O2C00P, CS04O2C00P, CS05O2C00P and CS06O2C00P species in full-volatility emission inventory used in the 2D-VBS mechanism where numbers after CS indicate the negative (M) or positive (0) ${\text{log}}_{10}\left(C_{i}^{*}\left[\mu \text{g}{\;\text{m}}^{-3}\right]\right)$ value and the number after 2C means 10 × nO : nC (e.g., CS05O2C00P is $C_{i}^{*}=10^{-5}\;\mu \text{g}{\;\text{m}}^{-3}$ with nO : nC = 0).

For oxygenated L/S/IVOC, the species in 2D-VBS mechanism were lumped into 15 CRACMM mechanism species, spanning $C_{i}^{*}$ values of 10^−2^ to 10^6^ μg m^−3^ and nO : nC of 0.1 to 0.8: ROCN2OXY2, ROCN2OXY4, ROCN2OXY8, ROCN1OXY1, ROCN1OXY3, ROCN1OXY6, ROCP0OXY2, ROCP0OXY4, ROCP1OXY1, ROCP1OXY3, ROCP2OXY2, ROCP3OXY2, ROCP4OXY2, ROCP5OXY1, and ROCP6OXY1. 2D-VBS products of known nC and nO were mapped to the available CRACMM model species, first by interpolating to the two nearest species in nO : nC space, and then to the two nearest species ${\text{log}}_{10}\left(C_{i}^{*}\right)$ points.

Since neither Saprc07tic_ae7i nor CB6r3_ae7 includes a representation of full-volatility POA, only CRACMM can utilize this comprehensive inventory. The methodology outlined by Chang et al. (2022) includes emissions from various sources, along with their corresponding profiles, volatility ranges, and emission amounts. The anthropogenic L/S/IVOC emission inventory using a volatility-binned approach with full coverage of both particle and gas phases is shown in Fig. S4. For traditional POA inventory, the POA emission amount was 2840 kt yr^−1^, while the new full-volatility emission inventory includes emissions of Low-Volatility Organic Compounds (LVOC) (1342 kt yr^−1^), SVOC (1169 kt yr^−1^), and IVOC (3939 kt yr^−1^), resulting in a total of 6450 kt yr^−1^. The new inventory fills a gap of 3610 kt yr^−1^ in L/S/IVOC emissions that were absent from the traditional inventory. To thoroughly evaluate CRACMM and compare it with CB6r3_ae7 and Saprc07tic_ae7i, four simulation scenarios were designed, as shown in Table 1.

In the CMAQ model, a potential combustion SOA (pcSOA) species is introduced to compensate for the fraction of SOA formed from the oxidation of combustion-related organic compounds that are not explicitly represented in the model (Murphy et al., 2017). Traditional chemical mechanisms often do not include SVOCs and IVOCs emitted from combustion sources, nor their associated oxidation pathways, which leads to a systematic underestimation of SOA levels in model simulations. To address this issue, CMAQ has incorporated the pcSOA species into the organic aerosol module since version 5.2, providing an empirical representation of this missing combustion-related SOA component (Murphy et al., 2017). This treatment is primarily intended for anthropogenic combustion sources, such as motor vehicle emissions, industrial combustion, and biomass burning. Because the CRACMM species framework explicitly accounts for multigenerational oxidation processes across different volatility ranges of VOCs, the empirical anthropogenic SOA source (pcSOA) implemented in the CB6r3_ae7 and Saprc07tic_ae7i mechanisms was turned off in all simulations to avoid double counting with the CRACMMv1.0 mechanism.

### 2.3 Observational data and model performance evaluation

Hourly concentrations of PM_2.5_ at national monitoring stations were obtained from the China National Environmental Monitoring Centre (https://air.cnemc.cn:18007/, last access: 26 March 2026), which were then used to evaluate model performance. Field observational data of PM_2.5_ chemical components including ${\text{NO}}_{3}^{-}$ (nitrate), ${\text{SO}}_{4}^{2-},{\text{NH}}_{4}^{+}$ (ammonium), OC (organic carbon), and EC (elemental carbon) at six super monitoring station sites were collected, as detailed in Fig. 1 and Table S9. Missing observation periods were excluded from the analysis. Model performance was assessed using well-established statistical metrics, including the R, MB, NMB, root mean square error (RMSE), normalized mean error (NME), and index of agreement (IOA). The formulas for each individual parameter are presented in Table S10. In these equations, ${\overset{‾}{C}}_{\text{m}}$ and ${\overset{‾}{C}}_{\text{o}}$ represent the mean modeled and observed concentrations over all samples, respectively; *C*_m_ and *C*_o_ denote the modeled and observed values for the *i*th sample; and *N* is the total number of valid samples. A combined analysis of these statistical indicators enables a comprehensive assessment of model performance and reliability, providing a basis for further model refinement and interpretation of the simulation results.

## 3 Results and discussion

### 3.1 Overview of CMAQ-CRACMM model performance evaluation on PM_2.5_

**Figure 2.**
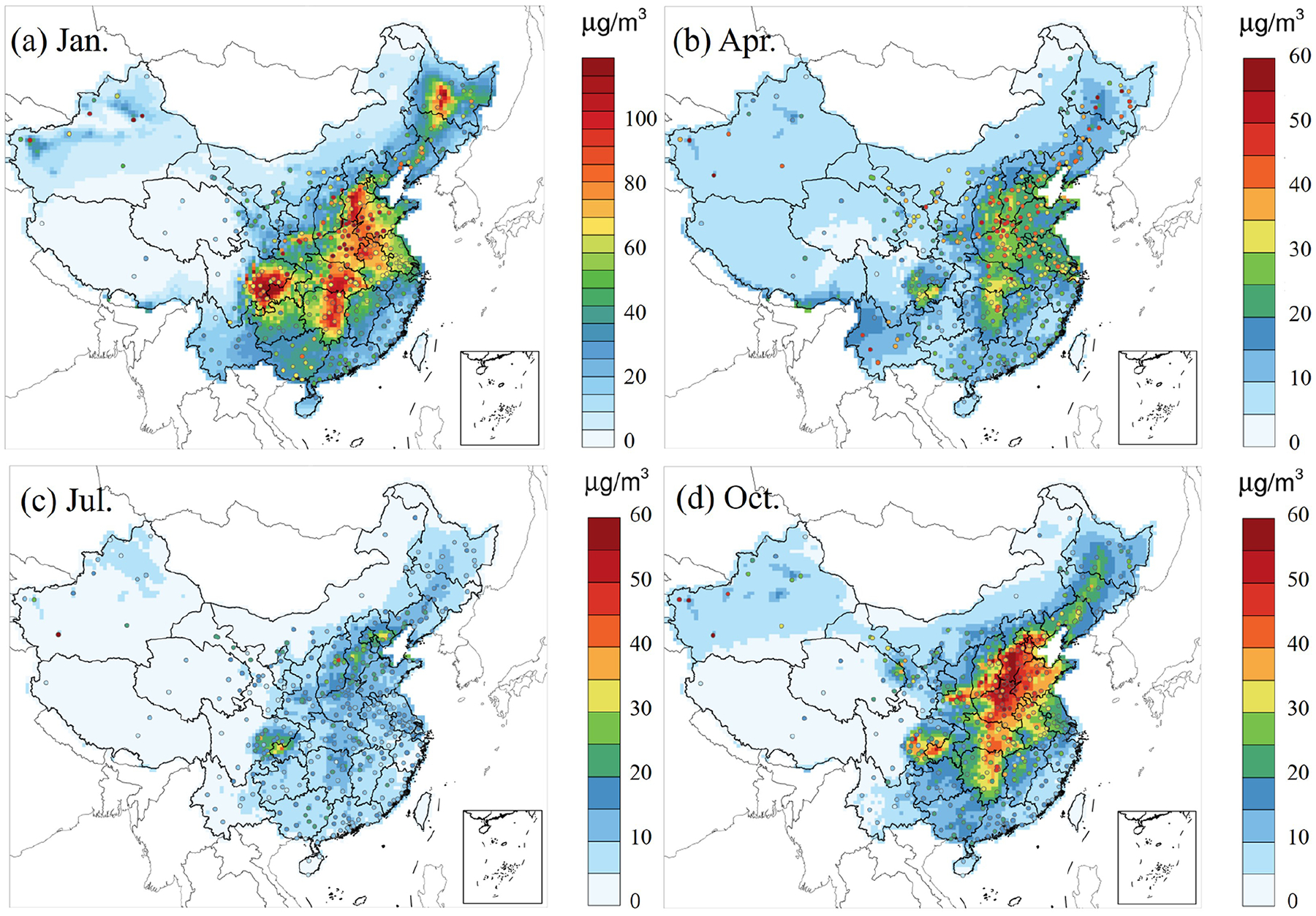
Monthly average PM_2.5_ concentrations predicted (raster) by CRACMM and observed (dots) in 2021 using the full-volatility emission inventory. Note that the color scale for panel **(a)** differs from panels **(b)**–**(d)** to highlight variations in the data.

Figure 2 depicts the spatial distribution of observed (dots) and simulated PM_2.5_ concentrations for January, April, July, and October 2021, based on the CRACMM model with the full-volatility inventory. In January (Fig. 2a), PM_2.5_ concentrations range from 5 to over 100 μg m^−3^, with the highest values concentrated in the NCP and parts of the SCB. These elevated levels are primarily driven by relatively higher anthropogenic emissions and stagnant meteorological conditions typical of winter. In April (Fig. 2b), concentrations have significantly decreased, ranging from 5 to 40 μg m^−3^, with the most notable reductions observed in northern regions. During July (Fig. 2c), PM_2.5_ concentrations were at their lowest, typically ranging from 0 to 40 μg m^−3^. This decline is mainly attributable to the increased precipitation which washed out pollutants, in the southern and eastern parts of China compared to other months (Fig. S5). In addition, higher planetary boundary layer (PBL) heights during the warm season in the south, particularly in the YRD region of China than other months (Fig. S6), enhancing atmospheric mixing and dilution, and led to the decrease in PM_2.5_ concentrations. In October (Fig. 2d), PM_2.5_ concentrations rise again, ranging from 5 to 60 μg m^−3^, with the highest concentrations observed in the NCP and along the eastern coastal regions, which is attributed to the heating in later autumn and unfavorable meteorological conditions. Overall, monthly variations in PM_2.5_ concentrations are primarily driven by meteorological conditions and the distribution of emission sources.

**Figure 3.**
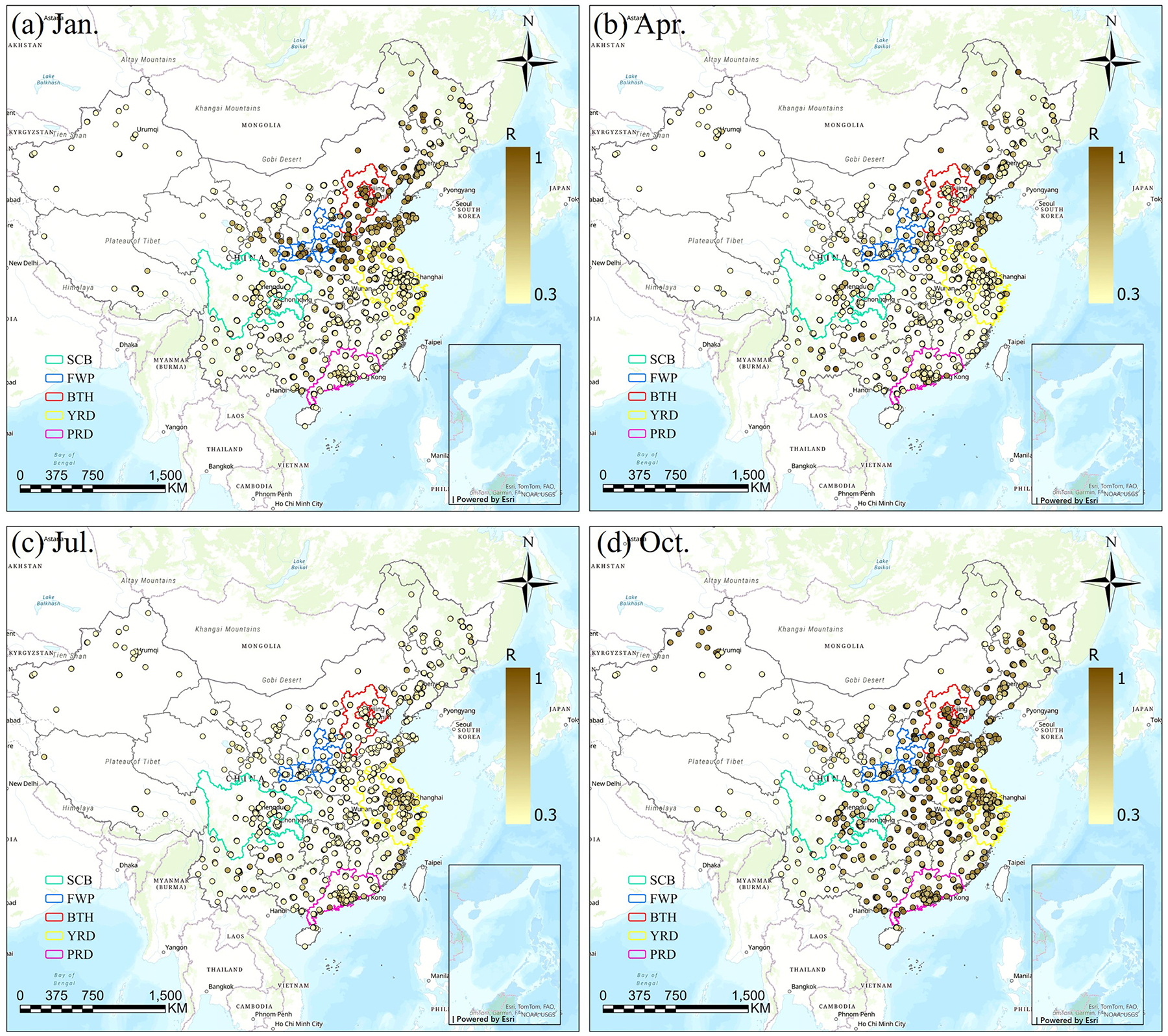
*R* values between predicted and observed PM_2.5_ concentrations using CRACMM with the full-volatility emission inventory for January, April, July, and October of 2021 (Esri, TomTom, FAO, Garmin, NOAA, USGS | Powered by Esri).

**Figure 4.**
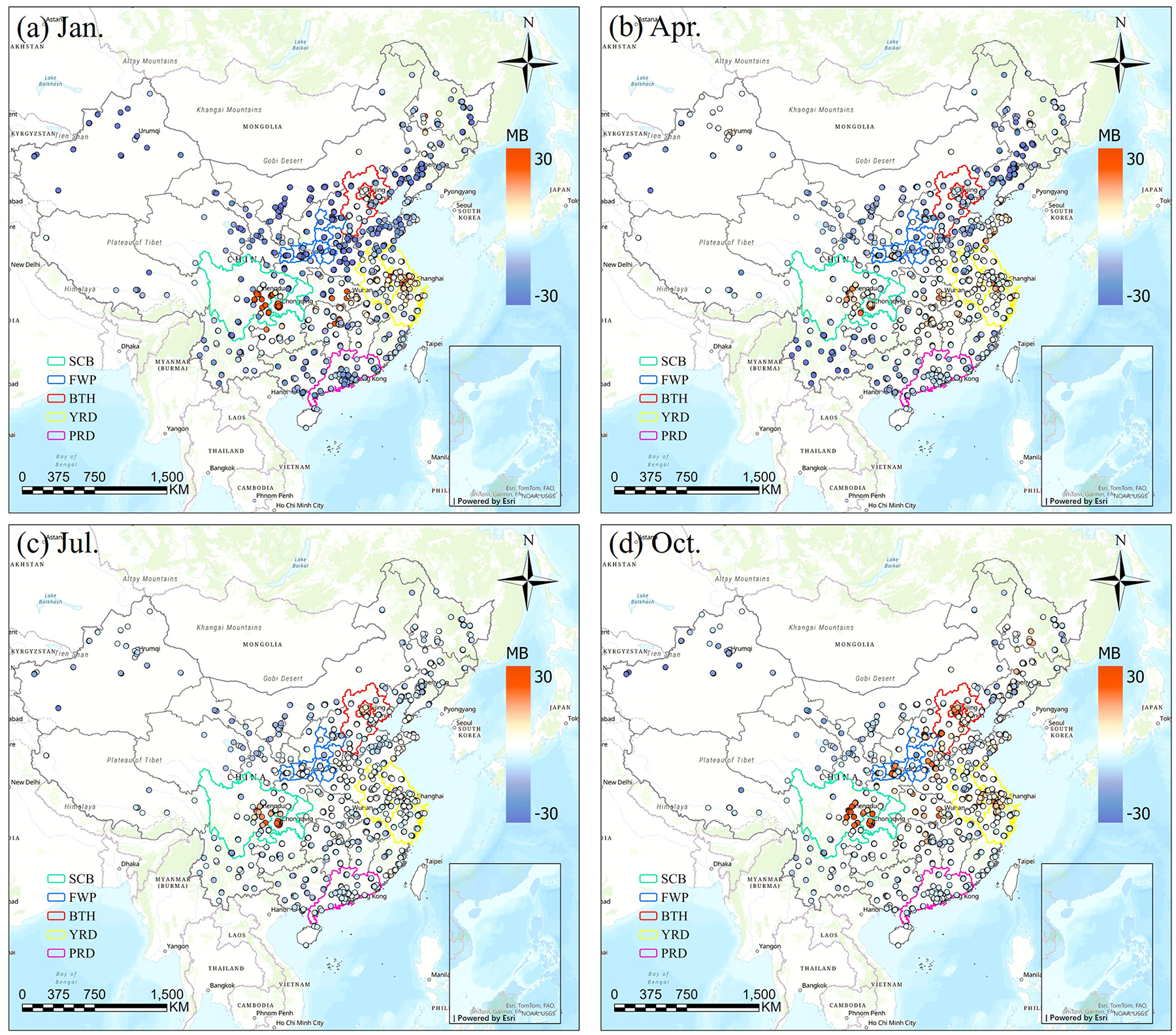
The MB values between predicted and observed PM_2.5_ concentrations using CRACMM with full-volatility inventory for January, April, July, and October of 2021 (Esri, TomTom, FAO, Garmin, NOAA, USGS | Powered by Esri).

The performance of the CMAQ model in simulating hourly PM_2.5_ concentrations was evaluated by comparing the model outputs with observations from national monitoring sites. In January, CRACMM shows higher *R* value (*R* > 0.7) over northern and eastern China (e.g., BTH, FWP), whereas lower *R* values (approximately 0.4) are found in southern regions (e.g., PRD) (Fig. 3a). The model generally underestimates PM_2.5_ across most areas (Fig. 4a), except for the YRD and SCB regions, where positive biases occur at many sites. This spatial contrast is partly attributable to dust-related influences, although data from the major dust episode on 13–14 January were excluded from the monthly evaluation, the absence of explicit dust emissions and the associated complex meteorological conditions likely contributed to PM_2.5_ underestimation of up to ~ 30 μg m^−3^ in northern China. Results for April (Figs. 3b and 4b) show generally good correlations in the eastern regions, while several monitoring sites in the south exhibit lower *R* values. Compared to January, the MB is less pronounced. April also experiences dust storm events. In July, *R* values decline across all regions relative to January and April, and most stations exhibit relatively small MB values (Figs. 3c and 4c). The strong influence of temperature and solar radiation on photochemical processes during summer may result in more pronounced diurnal variations in chemical composition, making the simulation of chemical processes more challenging (Seinfeld and Pandis, 1998). Moreover, the chemical mechanisms may inadequately capture non-linear interactions and the influence of SOA (Harrison et al., 2022), further reducing the correlation. Additionally, synoptic-scale variations can also affect the spatial distribution and concentration of key atmospheric species (Zhu et al., 2023). *R* values are improved in October (Figs. 3d and 4d), with 90 % of the sites achieving *R* values of 0.8 and the MB is around 10 μg m^−3^ with higher evaluation in SCB and BTH regions. Overall, wintertime observed peaks generally are underestimated and lower summertime observed values generally are well captured. The model demonstrates strong performance in January and October, characterized by higher correlations and smaller biases. However, it had weaker performance in April and July with lower correlations.

Figures S7 and S8 compare the *R* and MB values between CRACMM (with the full-volatility inventory) and CB6r3_ae7 (with the traditional inventory). In January, CRACMM demonstrates notable improvements in *R* values at several sites in the PRD and YRD regions, with increases ranging from 0.2 to 0.4 (Fig. S7a), while changes at most other sites remain relatively minor (0–0.1). Regarding MB, the most pronounced differences also occur in January: some locations in the BTH and YRD regions show higher MB values – up to 10 μg m^−3^ – with CRACMM, whereas other sites display reduced MB values (Fig. S8a). In April (Figs. S7b and S8b), CRACMM achieves higher *R* values (0–0.2) at certain sites in the YRD, while slightly lower correlations (0–0.16) are observed in the PRD compared to CB6r3_ae7. MB values remain elevated in the SCB and parts of the YRD region for CRACMM. For July (Figs. S7c and S8c), *R* values from CRACMM are generally comparable to those from CB6r3_ae7. However, MB values tend to decrease across most regions, indicating a potential improvement in bias performance during summer. In October, CRACMM shows moderate increases in *R* values – by approximately 0.1 at most sites (Fig. S7d). MB values are lower in the PRD region but higher in the FWP region compared to CB6r3_ae7 (Fig. S8d).

In evaluating the CMAQ model’s performance for hourly PM_2.5_ concentrations, CRACMM generally shows good correlations with observed data in January and October. However, discrepancies arise in April, likely due to chemical conditions such as dust storms, springtime dust events are frequently observed in China, particularly in northern regions, driven by strong surface winds and synoptic-scale transport (Huo et al., 2025). For July, larger discrepancies are likely associated with more complex meteorological conditions, as reflected by the relatively poor performance of wind speed, particularly in the SCB region. In contrast, the simulation performance of relative humidity and temperature shows only minor differences relative to the other three months (Tables S11–S13). In addition, photochemical activity typically peaks in the summer months (June–August) due to stronger solar radiation and higher temperatures (Gu et al., 2022), which further exacerbates these discrepancies.

The model tends to underestimate peak PM_2.5_ concentrations during winter but captures lower summer concentrations more accurately. Comparisons between CRACMM (with the full-volatility inventory) and CB6r3_ae7 (using the traditional inventory) highlight improvements in *R* values in the PRD and parts of the YRD regions in January for CRACMM, although performance declines in July.

The simulation performance of PM_2.5_ components in six selected cities using CRACMM with the full-volatility inventory was evaluated. Some observations were from the previous study (Wang et al., 2024). Tables S14–S17 summarize the statistical values of PM_2.5_ components, including ${\text{NO}}_{3}^{-},{\text{SO}}_{4}^{2-},{\text{NH}}_{4}^{+}$, OC, and EC, for the four selected months. Figures S13–S16 demonstrate that CRACMM effectively captured the overall peaks and troughs of observed PM_2.5_ concentrations in January. The model also successfully simulated the heavy pollution period from 20–25 January in Taiyuan, with results similar to our previous study (Wang et al., 2024). The three ions were well simulated in both Changzhou and Pudong in the YRD region, particularly in Changzhou, where the overall trends of OC and EC showed strong consistency with observations. However, for EC in Pudong, the model struggled to capture the hourly peak values accurately.

As shown in Table 2, the results of performance evaluation based on observations from over 1300 monitoring sites across China for CRACMM shows that the correlation metrics (*R* and IOA) met the recommended benchmark (Huang et al., 2021b) in October, while they fell short of the benchmark in January, April, and July. In contrast, the bias metrics (NMB and NME) satisfied the recommended benchmark across all four representative months, indicating that the model performed well in controlling overall bias. The evaluation metrics for different regions and months are provided in Tables S18–S21. Previous studies have reported similar *R* values. For example, the evaluation results of PM_2.5_ simulations using CMAQ over five key regions, which were the same five regions used in our study, showed that the R were generally below 0.6 in July (Huang et al., 2024). In addition, a multi-model intercomparison study involving CAMx6.2, CAMx7.1, CMAQv5.0.2 and CMAQv5.3.2 reported *R* values of 0.58, 0.55, 0.60, and 0.39, respectively, for mean PM_2.5_ concentrations in China during 2014–2017. The CAMx6.2, CAMx7.1, and CMAQv5.0.2 simulations covered the entire country, whereas CMAQv5.3.2 was evaluated over eastern China (Meng et al., 2026). The heatmaps in Fig. S9 illustrate the variations in *R* values across the five key regions – YRD, SCB, PRD, FWP, and BTH – for the three chemical mechanisms over four representative months. Corresponding heatmaps for MB, NMB and RMSE are presented in Figs. S10, S11 and S12, respectively.

**Figure 5.**
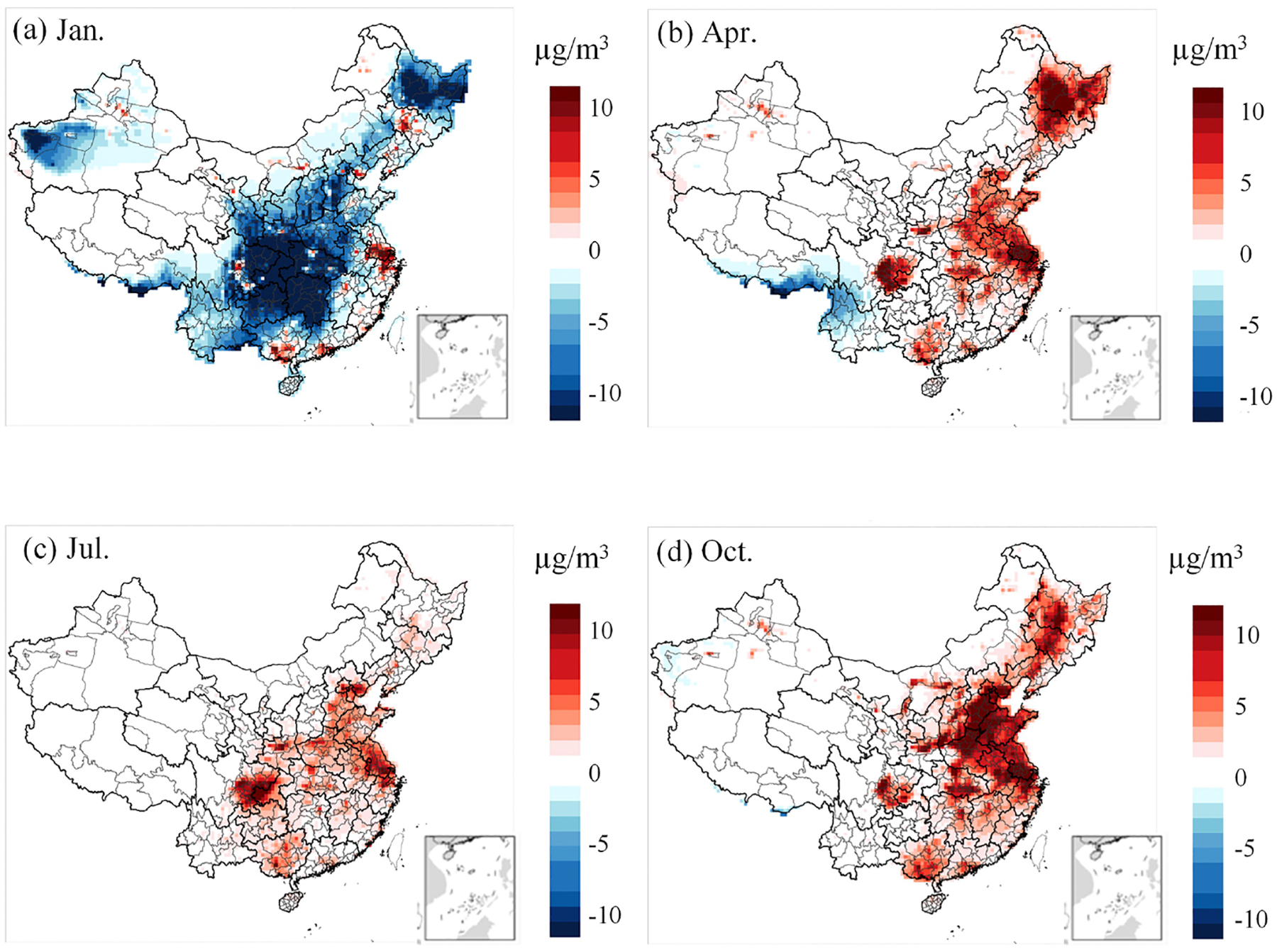
Differences in model-predicted PM_2.5_ concentrations between CRACMM (full-volatility inventory) and CB6r3_ae7.

In the YRD region, all mechanisms show relatively stable *R* values across the four months (Fig. S9). The CRACMM simulation using the traditional inventory consistently results in lower MB (Fig. S10) and NMB (Fig. S11) values compared to CB6r3_ae7 and Saprc07tic_ae7i throughout the year, RMSE values range from for 0–2 μg m^−3^ (Fig. S12). When the full-volatility inventory is incorporated, MB improves in April and July but worsens in January and October. Similarly, NMB values indicate higher modeled concentrations in all four months with the full-volatility inventory compared to the traditional one. This trend is consistent with Fig. 5, where the YRD region shows higher modeled concentrations from CRACMM using the full-volatility inventory than from both CB6r3_ae7 and Saprc07tic_ae7i across all months.

In the SCB region, *R* values remain relatively consistent across the three mechanisms (Fig. S9a–c). A slight improvement is observed in July, where *R* increases from 0.22 with the traditional inventory to 0.27 with the full-volatility inventory (Fig. S9d). However, in October, *R* decreases from 0.62 to 0.56 after switching to the full-volatility inventory. The increase in *R* values in July suggests that the traditional inventory may underestimate key precursors (e.g., S/IVOCs), while the full-volatility inventory better captures these species active at higher temperatures, improving model–observation agreement. In contrast, the October decrease in *R* may reflect uncertainties in representing some gas- or particle-phase organics under cooler conditions. Regarding MB, CRACMM generally shows reduced values in most months when using the traditional inventory, except for April. With the full-volatility inventory, MB decreases further in October, while slight increases are observed in the other months. The trends in NMB follow a similar pattern to those in MB. It could be due to overestimation of certain intermediate- or low-volatility species under specific conditions.

In the PRD region, CRACMM exhibits notable performance improvements in January. As shown in Fig. S9a–c, the *R* value increases from 0.20 with CB6r3_ae7 and Saprc07tic_ae7i to 0.35 when using CRACMM with the traditional POA inventory. When the full-volatility inventory is applied (Fig. S9d), the *R* value further increases to 0.50. Concurrently, the MB improves significantly, decreasing from −19.6 to −11.8 μg m^−3^, and the NMB is reduced from −43 % to −26 %. These results indicate that CRACMM, particularly with the full-volatility inventory, achieves both higher correlation and lower bias for PM_2.5_ simulations in the PRD region during January. Under the traditional POA inventory, CRACMM tends to underestimate PM_2.5_ concentrations in PRD during January (Fig. S8a). However, after switching to the full-volatility inventory, simulated concentrations exceed those predicted by CB6r3_ae7 (Fig. 5a), primarily due to increased contributions from SOA (Fig. 8b). In comparison, during April and July, the CRACMM simulations using the traditional emissions inventory showed lower *R* values than those of CB6r3_ae7 and Saprc07tic_ae7i, and the correlation further declined when using the full-volatility emissions inventory. Although CRACMM features a more comprehensive design for gas-phase oxidation mechanisms, the intense photochemical activity in July and the rapid oxidation of high concentrations of IVOC precursors may have introduced more complex SOA formation pathways and product distributions, thereby increasing modeling uncertainties and weakening the agreement with observations. By October, model performance had improved. Across all months, CRACMM combined with the full-volatility emissions inventory consistently outperformed the other mechanisms in terms of MB and NMB, highlighting the critical role of this inventory in addressing the underestimation of PM_2.5_ associated with traditional POA treatment.

In the FWP region, both the *R* (Fig. S9a–c) and MB (Fig. S9a–c) values show minimal variation across the three mechanisms, indicating limited sensitivity to the chemical mechanism alone. However, notable changes in MB and NMB are observed with the incorporation of the full-volatility inventory in April, July and October, which align with the higher PM_2.5_ concentrations shown in Fig. 5, compared to CB6r3_ae7. In April and July, MB values shift from −12.7 and −5.6 to −2.4 and 1.2 μg m^−3^, respectively, with NMB showing a similar trend. These changes suggest an improvement in model agreement when the full-volatility inventory is employed. In contrast, both MB and NMB increase in October, indicating that the full-volatility inventory leads to higher simulated concentrations during this month.

In the BTH region, The *R* (Fig. S9a–c) and MB (Fig. S10a–c) values remain largely consistent across the three mechanisms. CRACMM with the traditional POA inventory shows a decrease in *R* values from 0.44 to 0.38 compared to CB6r3_ae7 in July. After incorporating the full-volatility inventory, the *R* in BTH experiences a more significant drop, falling to 0.24. Notably, MB and NMB indicate that the modeled results are lower for CRACMM with the traditional POA inventory in January, while they are higher in the other months. Additionally, BTH exhibited the highest IVOC emissions in the inventory (Chang et al., 2022), and uncertainties in emission estimates may have further contributed to this result.

Overall, the differences in *R* and MB values across the three chemical mechanisms are relatively small when the traditional POA inventory is used. However, for CRACMM, the MB in January indicates a stronger underestimation, primarily due to differences in POA (as shown in Fig. 8a), leading to lower modeled concentrations compared to CB6r3_ae7 and Saprc07tic_ae7i. With the incorporation of the full-volatility inventory, the MB shifts toward higher modeled concentrations in the subsequent three months. Notably, more pronounced differences are observed in January in the PRD region and in July in the BTH region.

### 3.2 Comparisons of model predicted PM_2.5_ between CRACMM and other mechanisms

**Figure 6.**
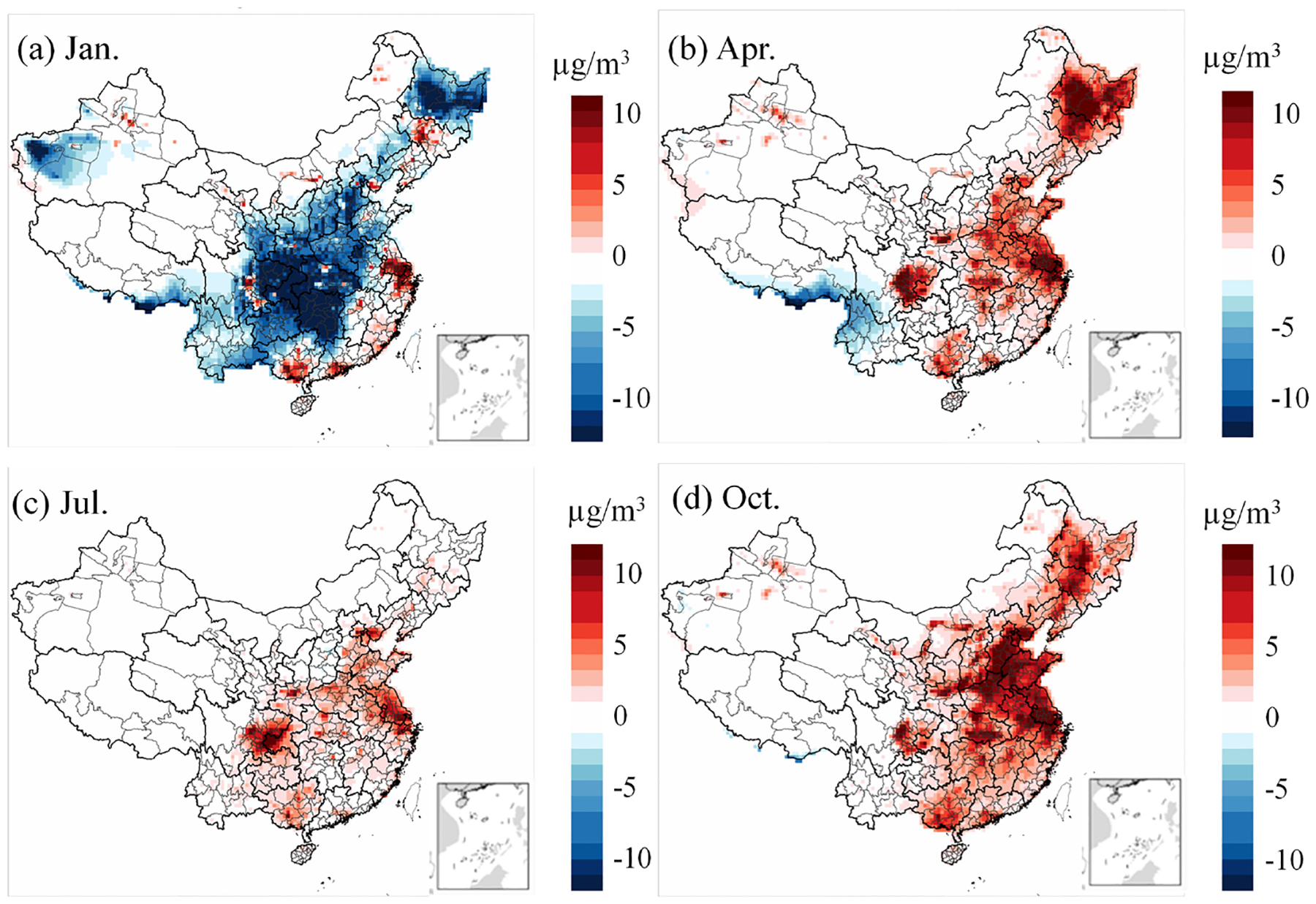
Differences in model-predicted PM_2.5_ concentrations between CRACMM (full-volatility inventory) and Saprc07tic_ae7i.

Figures 5 and 6 illustrate the differences in model outputs between CRACMM with full-volatility inventory and CB6r3_ae7, as well as between CRACMM and Saprc07tic_ae7i. In January, CRACMM predicts lower PM_2.5_ concentrations across central and northern China compared to both CB6r3_ae7 (Fig. 5a) and Saprc07tic_ae7i (Fig. 6a), with the differences – up to 10 μg m^−3^ – observed in central and north of China. While CRACMM simulates higher PM_2.5_ concentrations in the PRD and YRD regions. For the remaining months – April, July, and October – CRACMM with full-volatility inventory generally predicts higher PM_2.5_ levels than the other two mechanisms (Figs. 5b–d, 6b–d). When CRACMM and CB6r3_ae7 are configured with the traditional POA inventory, as shown in Fig. S17, the differences of PM_2.5_ concentrations are reduced in April, July, and October compared with full-volatility POA inventory. But CRACMM still predicts lower PM_2.5_ levels than CB6r3_ae7 in January. A likely explanation is that the lower photochemical activity leads to reduced SOA formation, as the enhanced SOA pathways are less active during the winter months.

### 3.3 Comparisons of model predicted PM_2.5_ chemical components between CRACMM and other mechanisms

Analysis of Figs. 5, 6, and S17 indicates that the most significant differences among the model simulations occur in January and October, whereas the discrepancies between chemical mechanisms are substantially smaller in April and July – likely due to the overall lower pollutant concentrations during these months, which may reduce the sensitivity to mechanistic differences. Consequently, the subsequent analysis focuses on a detailed comparison of PM_2.5_ component variations between CRACMM and CB6r3_ae7 for January and October.

#### 3.3.1 Inorganic aerosol

**Figure 7.**
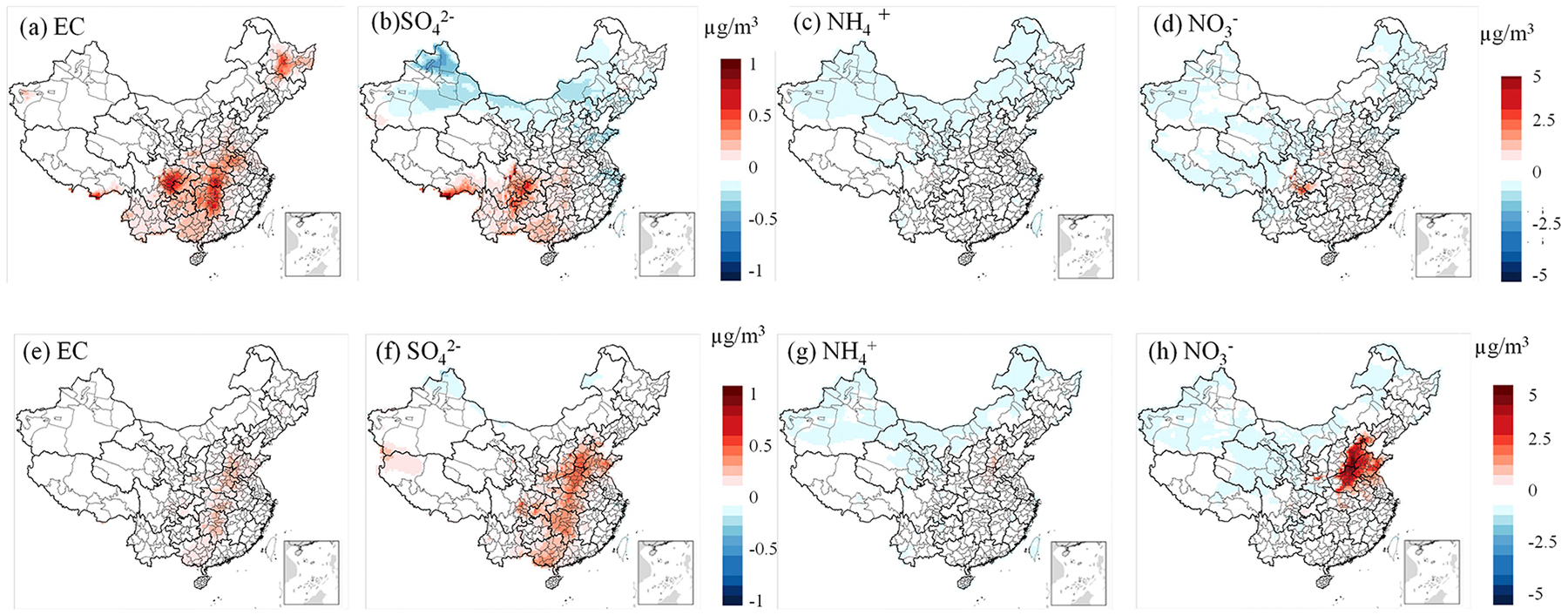
Differences in model-predicted PM_2.5_ components – **(a)** EC, **(b)** ${\text{SO}}_{4}^{2-}$, **(c)** ${\text{NH}}_{4}^{+}$, **(d)** ${\text{NO}}_{3}^{-}$ for January, and **(e)** EC, **(f)** ${\text{SO}}_{4}^{2-}$, **(g)** ${\text{NH}}_{4}^{+}$, **(h)** ${\text{NO}}_{3}^{-}$ for October – between CRACMM (with full-volatility inventory) and CB6r3_ae7. Subplots **(a)–(b)** and **(e)–(f)** are plotted using the same color scale, as are subplots **(c)–(d)** and **(g)–(h)**.

${\text{SO}}_{4}^{2-},{\text{NO}}_{3}^{-}$, and ${\text{NH}}_{4}^{+}$ are the dominant secondary inorganic components in PM_2.5_. Nitrogen dioxide (NO_2_) and sulfur dioxide (SO_2_) can fully dissolve into cloud water or aerosol liquid phases and subsequently oxidize to form nitrate and sulfate. Ammonium salts are produced through the neutralization reactions of these acidic species with atmospheric ammonia (NH_3_). EC primarily originates from the incomplete combustion of carbonaceous fuels, especially under oxygen-limited conditions. It is commonly emitted from sources such as vehicle exhaust, industrial combustion, and biomass burning. From a chemical mechanism perspective, CRACMM retains the inorganic chemistry framework of RACM2 but incorporates updated rate constants for several reactions. Specifically, the rate expressions for 26 inorganic reactions were revised in CRACMM compared to RACM2 (Pye et al., 2023b). Overall, differences in inorganic component predictions among CRACMM, CB6r3_ae7, and Saprc07tic_ae7i are relatively minor. As shown in Fig. 7, predicted concentrations of major inorganic species in January and October are comparable between CRACMM and CB6r3_ae7, with differences ranging from −1 to 1 μg m^−3^ for EC and ${\text{SO}}_{4}^{2-}$, and −5 to 5 μg m^−3^ for ${\text{NH}}_{4}^{+}$ and ${\text{NO}}_{3}^{-}$. These results suggest that the variation in simulated inorganic aerosol concentrations is only marginally affected by differences in the inorganic chemistry schemes.

#### 3.3.2 Organic aerosol

**Figure 8.**
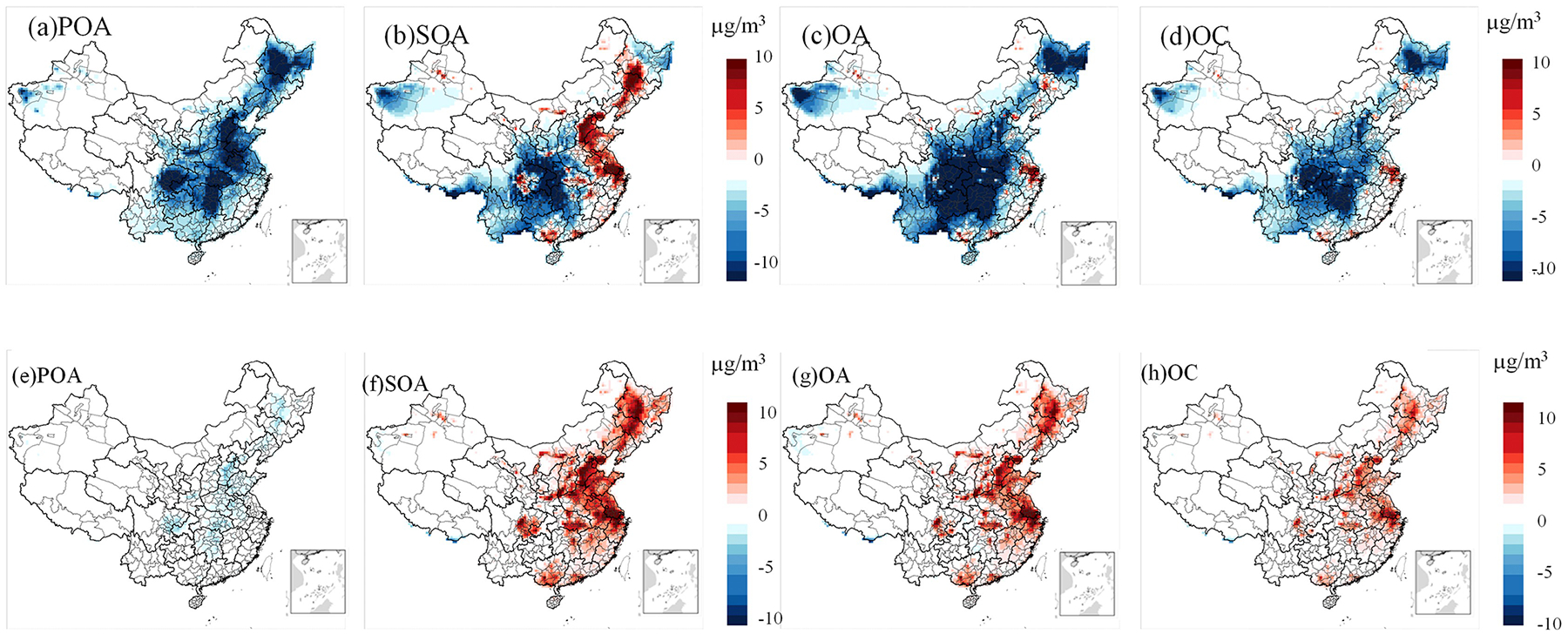
Differences in model-predicted PM_2.5_ components – **(a)** POA, **(b)** SOA, **(c)** OA, and **(d)** OC for January, and **(e)** POA, **(f)** SOA, **(g)** OA, and **(h)** OC for October – between CRACMM (with full-volatility inventory) and CB6r3_ae7.

In January, CB6r3_ae7 consistently predicts higher POA concentrations than CRACMM under both the traditional and full-volatility inventory configurations, with the most pronounced differences occurring in east China (Figs. 8a and S18a). In CRACMM, POA aging is represented using a modified 2D-VBS framework (Murphy et al., 2017), where $C_{i}^{*}$ range from 10^−2^ to 10^3^ μg m^−3^, $C_{i}^{*}$ represents the effective saturation concentration, which characterizes the volatility of organic compounds and influences gas-particle partitioning. A significant portion of the alkane-like L/SVOC mass contributing to ambient OA comes from the direct emissions of low-volatility species (e.g., AROCN2ALK, AROCN1ALK, AROCP0ALK, AROCP1ALK, AROCP2ALK, AROCP3ALK, where the numbers indicate negative (N) or positive (P) ${\text{log}}_{10}\left(C_{i}^{*}\left[\mu \text{g}{\;\text{m}}^{-3}\right]\right)$ value. When species reside in the gas-phase as a vapor, it is prefixed with a “V” and when in the particle phase, a prefix “A” is used. For example, VROCP2ALK is an alkane-like vapor species with $C_{i}^{*}$ of 10^2^ μg m^−3^, and AROCP2ALK is a particulate species of the same volatility. and their oxidation products (e.g., AROCN2OXY2, AROCP0OXY2, AROCP1OXY1, AROCP2OXY2, AROCP3OXY2), these species follow a similar naming convention as the L/S/IVOC alkanes, where numbers after N and P indicate negative or positive ${\text{log}}_{10}\left(C_{i}^{*}\right)$ value and the value ends in 10 × nO : nC (e.g., ROCN2OXY2 is $C_{i}^{*}=10^{-2}\;\mu \text{g}{\;\text{m}}^{-3}$ with nO : nC = 0.2). By contrast, CB6r3_ae7 adopts a semi-volatile POA approach in which primary emissions (e.g., LVPO1, SVPO1–3, IVPO1) and their oxidation products (e.g., (LVOO1, LVOO2, SVOO1, SVOO2, SVOO3) partition between gas and particle phases across a $C_{i}^{*}$ range of 10^−1^ to 10^3^ μg m^−3^. This framework aligns with the 1.5D-VBS scheme proposed by Koo et al. (2014). Details of the POA species and their properties are provided in Table S7. The most significant differences in simulated POA concentrations occur in January, likely due to enhanced partitioning of SVOC to the particle phase under low wintertime temperatures. Additionally, differences in multigenerational oxidation aging and volatility treatment between the two mechanisms contribute to the simulation discrepancies. In October, although POA concentrations in CRACMM remain lower than in CB6r3_ae7, the difference is less pronounced compared to January for both POA inventory (Figs. 8e and S12e).

To better understand the drivers of SOA formation in the two mechanisms, we analyzed the spatial distribution of SOA concentrations under both traditional and full-volatility POA inventories. In January, CRACMM predicts higher SOA levels in the BTH and parts of the SCB regions compared to CB6r3_ae7 when using the traditional POA inventory (Fig. S18b). Under the full-volatility inventory, CRACMM also shows increased SOA concentrations in the YRD and PRD regions (Fig. 8b). These increases correspond with high IVOC emissions in BTH and YRD, consistent with the spatial patterns reported by Chang et al. (2022), although their data reflect monthly averages across January and July. The SOA enhancement in YRD and PRD under the full-volatility inventory highlights the critical role of IVOC emissions in these areas. In CB6r3_ae7, SOA is primarily formed from the oxidation of traditional VOC sources, such as isoprene, monoterpenes, sesquiterpenes, benzene, toluene, xylene, alkanes, and PAHs (Carlton et al., 2010; Pye and Pouliot, 2012).

In contrast, CRACMM incorporates additional SOA precursor systems, including phenol and aromatic diols, pinon aldehyde, oxygenated IVOCs, furanone, and other compounds. As a result, in regions with elevated anthropogenic emissions, CRACMM generally simulates higher SOA concentrations. However, in the SCB region, SOA levels remain lower, possibly due to the reduced reactivity of these new precursors under the lower ambient temperatures typical of this region. In terms of overall OA concentrations, CRACMM generally predicts lower values than CB6r3_ae7 across most regions, except for some parts of YRD region (Figs. 8c and S18c). The spatial distribution of OC is similar to that of OA, with CRACMM also showing lower concentrations than CB6r3_ae7 (Figs. 8d and S18d).

In October (Figs. 8f–h and S18e–h), SOA remains the dominant contributor to the differences in PM_2.5_ concentrations. Under the full-volatility inventory, CRACMM predicts significantly higher SOA concentrations compared to CB6r3_ae7 (Fig. 8f), resulting in elevated OA levels (Fig. 8g). The spatial pattern of OC concentrations (Fig. 8h) closely resembles that of OA. When the traditional POA inventory is applied, the differences in SOA, OC, and OA concentrations between the two mechanisms are minimal (Fig. S18e–h). The most pronounced increases with the full-volatility inventory are observed in the YRD, PRD, and SCB regions, attributable to the inclusion of a more comprehensive set of SOA precursors.

Overall, in January, the primary differences between CRACMM and CB6r3_ae7 stem from lower POA concentrations in CRACMM, primarily due to semi-volatile partitioning and reduced aging of semi-volatile POA species at lower temperatures. SOA concentrations are elevated in eastern China but reduced over the SCB, reflecting both the slower oxidation of additional SOA precursors under winter conditions in the SCB and the greater availability of these precursors in the eastern region. In October, the key differences in model predictions are primarily driven by the POA inventory used. The full-volatility inventory yields higher SOA concentrations than the traditional inventory, largely due to the inclusion of L/S/IVOCs, which are efficient SOA precursors.

### 3.4 Sensitivity study on PM_2.5_ and SOA responses to changes of precursors

In this section, CMAQ simulations with emission perturbations are conducted to identify the key drivers of PM_2.5_ formation in January, when PM_2.5_ concentrations are notably high. A series of emission sensitivity simulations were performed within CMAQ to assess the role of precursor ROC systems in PM_2.5_ formation using CRACMM with the full-volatility inventory across China. These sensitivity simulations involved running zeroed emission scenarios for January (i.e., setting emissions of a specific chemical class or sector to zero) to examine how PM_2.5_ concentrations respond to changes in emissions. A subset of these sensitivity simulations was also conducted using the CB6r3_ae7 and Saprc07tic_ae7i mechanisms. A detailed list of all the zeroed emission simulations is provided in Tables 3 and S22. Due to the non-linear nature of PM_2.5_ production in response to ROC perturbations, these simulations offer an initial evaluation of how PM_2.5_ formation responds to reduced ROC emissions, providing valuable insights into how chemical systems behave under varying emission conditions in the three mechanisms.

**Figure 9.**
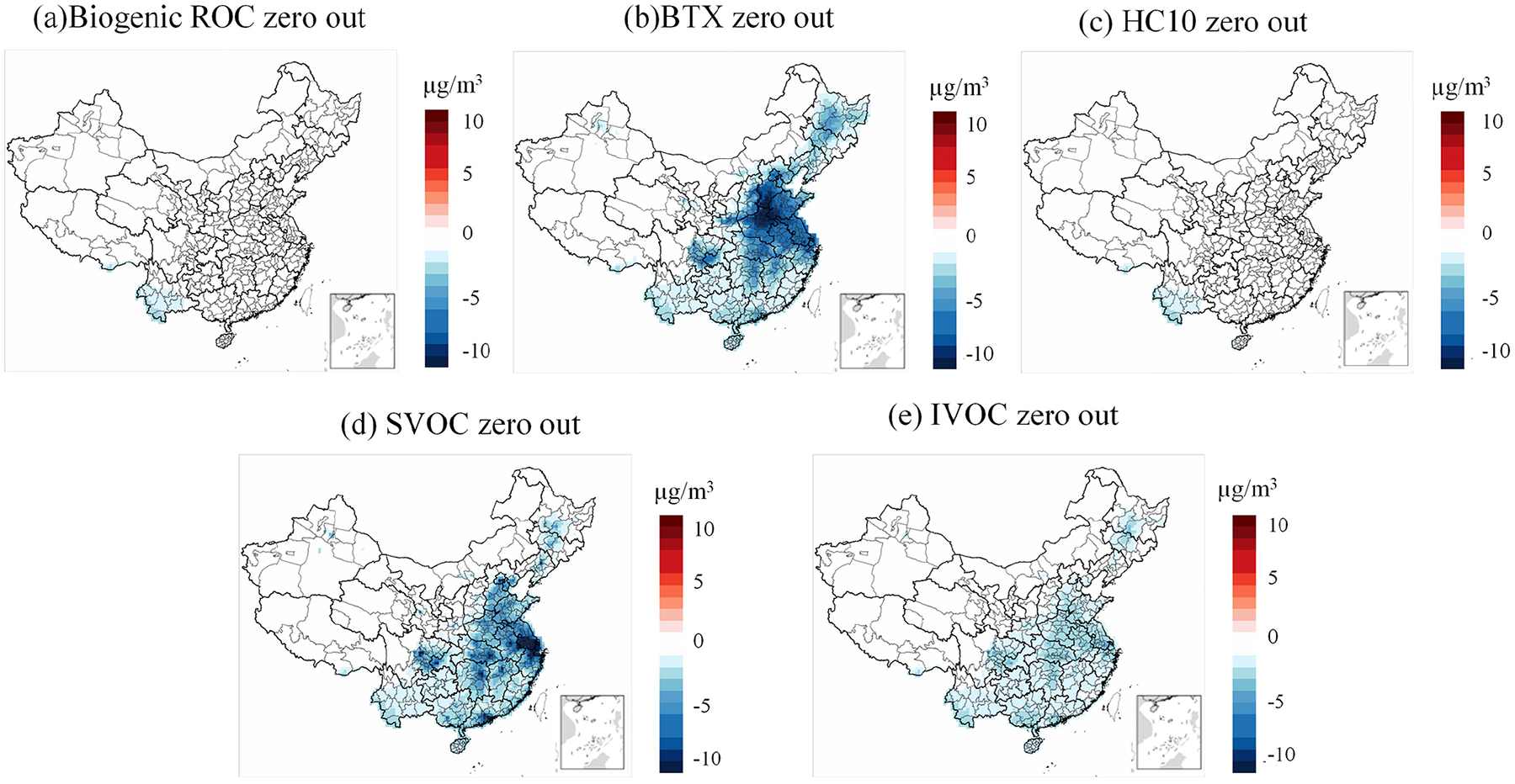
Changes in PM_2.5_ concentrations between each zero-out scenario and its corresponding base simulation: **(a)** biogenic ROC emission, **(b)** BTX emission, **(c)** HC10 emission, **(d)** SVOC emission, and **(e)** IVOC emission with CRACMM.

Figure 9 shows domain-wide differences in average PM_2.5_ concentrations between the base CRACMM simulation and a series of zeroed emission simulations. A similar spatial pattern in PM_2.5_ response was observed for zeroed biogenic and HC10 emissions (Fig. S19a, c in percentage and Fig. 9a, c in concentration), with CRACMM predicting a modest 1 % change in PM_2.5_ and less than 3 μg m^−3^ in many parts of China. This can be attributed to the low SOA yield by mass (0.09 g g^−1^) for HC10 compounds (Pye et al., 2023), and the generally low winter emissions of biogenic ROC, as shown in previous studies (Pye et al., 2023). Zeroing BTX emissions resulted in average PM_2.5_ concentration changes of −20 % to 0 % (Fig. S19b) and −10 to 0 μg m^−3^ (Fig. 9b), particularly in the YRD and BTH regions, where BTX emissions are highest. The high PM_2.5_ formation potential of BTX compounds is attributed to their overall emission abundance and high SOA yield by mass (~ 0.5 g g^−1^). Moreover, ozone levels in urban areas with significant BTX emissions also decrease in the BTX zero-out scenario (Fig. S20). This effect is particularly evident in the PRD region, where the reduction reaches up to 10 μg m^−3^. This aligns with the findings of Place et al. (2023). One factor is the removal of BTX emissions, which serve as precursors for SOA formation. The second factor is that zeroing BTX emissions leads to a decrease in O_3_, which weakens atmospheric oxidizing capacity and reduces SOA formation. However, it is important to note that Place’s study was conducted in summer.

**Figure 10.**
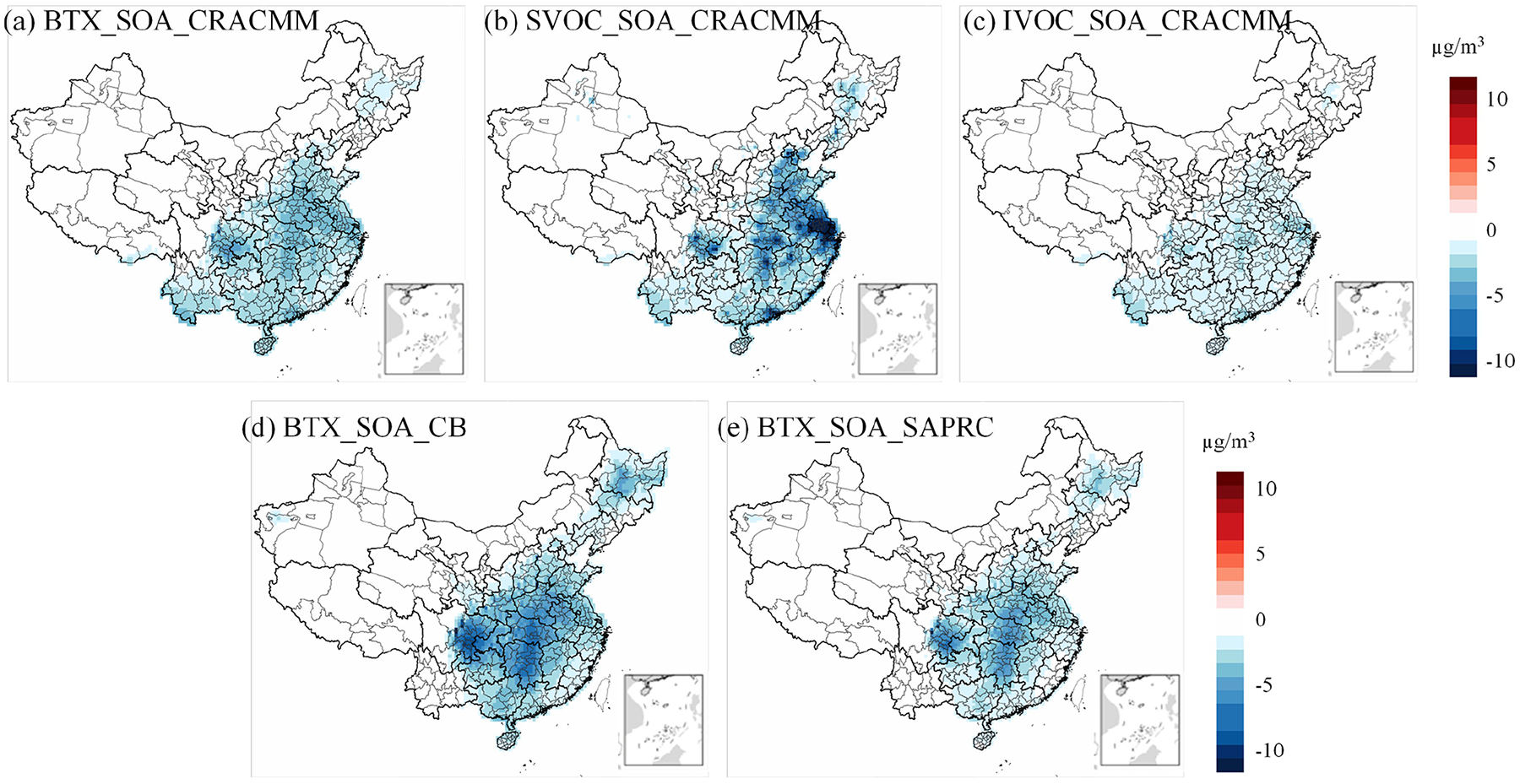
Changes in SOA concentrations between each zero-out scenario and its corresponding base simulation: **(a)** BTX emissions, **(b)** SVOC emissions, **(c)** IVOC emissions with CRACMM, **(d)** BTX emissions with CB6r3_ae7, **(e)** zeroed BTX emissions with Saprc07tic_ae6.

As shown in Fig. 10a, d, and e, the impact of zeroing BTX species on SOA formation for the three mechanisms accounts for only approximately 50 % of the total PM_2.5_ change observed in Figs. 9b, S16a and c. This suggests that the changes in PM_2.5_ concentrations resulting from the removal of BTX emissions are not solely due to SOA formation but may also involve other pathways or chemical processes. In contrast, the influence of S/IVOC species on PM_2.5_ concentrations in the CRACMM mechanism is primarily driven by SOA formation. This conclusion is supported by the spatial distribution and concentration differences shown in Figs. 9d, e, and 10b, c, which exhibit nearly identical patterns. These similarities indicate that the effects of S/IVOC emissions on PM_2.5_ are mainly driven by SOA production. The largest PM_2.5_ response was observed when emissions from SVOC sources were excluded from the simulation (Fig. S19d), primarily because SVOCs have the highest yield, exceeding 1.0 g g^−1^. The percentage changes in PM_2.5_ range from −40 % to 0 %, with a concentration reduction of more than −10 μg m^−3^ (Fig. 9d), particularly in the YRD region, where SVOC emissions are substantial. For IVOCs, the reduction is about −5 % across much of China, except in the western regions where IVOC emissions are very low (Fig. S19e), resulting in a reduction of less than −5 μg m^−3^ (Fig. 9e).

A similar ΔPM_2.5_ response in percentage (Fig. S21) and concentration change (Fig. S22) was observed when biogenic and BTX emissions were zeroed in simulations using CB6r3_ae7_ae7 and Saprc07tic_ae7i. However, the CRACMM simulation with zeroed biogenic emissions (Fig. 9a) showed a more pronounced and widespread decrease in PM_2.5_ compared to both CB6r3_ae7_ae7 and Saprc07tic_ae7i. This difference can be attributed to the inclusion of new S/IVOC species in MEGAN, which are not accounted for in the other two mechanisms. Additionally, zeroing BTX emissions had a greater impact in CB6r3_ae7, particularly in central China, compared to Saprc07tic_ae7i and CRACMM. A possible reason for this is that CRACMM reduces the number of lumped species in BTX and enhances the representation of aromatic IVOC species, such as single-ring aromatics ${\text{log}}_{10}\left(C_{i}^{*}\right)\approx 5$ (ROCP5ARO) and ${\text{log}}_{10}\left(C_{i}^{*}\right)\approx 6$ (ROCP6ARO). These species are included in CB6r3_ae7 under categories like *m*-xylene and other more reactive aromatics (XYM), as well as less reactive aromatics (XYE). As a result, CRACMM incorporates fewer species in BTX emissions compared to CB6r3_ae7.

### 3.5 Uncertainty Analysis and limitations

#### 3.5.1 Limitations of VOC Speciation Mapping

In the speciation mapping process, explicit species were directly mapped across the CRACMM, CB6r3_ae7, and Saprc07tic_ae7i mechanisms, as these species are explicitly represented and therefore allow for one-to-one mapping. Consequently, their spatial distributions were assumed to be identical across the three mechanisms, and uncertainties associated with their emission estimates were considered negligible. In contrast, lumped species present greater complexity due to differences in the VOC species included within each lumped mechanism species. Given that a direct one-to-one mapping between lumped species is not feasible, the mapping was performed by matching the dominant lumped VOC species across mechanisms based on their relative emission magnitudes. Overall, CRACMM incorporates a more comprehensive set of VOC species than either CB6r3_ae7 or Saprc07tic_ae7i. The total mapped emissions associated with each mechanism-specific inventory are summarized in Table S6. Nevertheless, uncertainties remain due to regional differences in emission profiles. In particular, the total emissions and source sector distributions of VOC species in Chinese emission inventories may differ from those represented in the NEI. Such discrepancies introduce additional uncertainty into the speciation mapping process.

#### 3.5.2 Uncertainty in Mapping L/S/IVOC Emissions

For the species which can be mapped on a one-to-one basis in Table S8, the associated uncertainty is assumed to be zero. For species mapping involving the same ${\text{log}}_{10}\left(C_{i}^{*}\right)$ values, such as VROCP1OXY1, VROCP0OXY4, and VROCN2OXY4, some uncertainty may be introduced due to the proximity in volatility space. Anthropogenic L/S/IVOCs emission inventories for China (Fig. S4) contains (a) particle-phase emissions with full-volatility coverage and (b) gas-phase emission inventories (Chang et al., 2022). Based on uncertainties of activity data and emission factors for each sector, the uncertainty of OA emissions can be quantified using a Monte Carlo method. According to Chang et al. (2022), the overall uncertainties at the 95 % confidence interval for LVOC, SVOC, IVOC and VOC are (−40 %, +43 %), (−35 %, 38 %), (−33 %, +33 %) and (−21 %, +28 %), respectively. The overall uncertainty for L/S/IVOC is (−25 %, +30 %). Uncertainties across sectors tend to partially offset each other, resulting in a total emission uncertainty that is often smaller than that of the individual sectors. S/IVOC emissions from domestic Volatile Chemical Products (VCPs) have the largest uncertainties (−81 %, +143 %), followed by open biomass burning (−58 %, +81 %) and industrial VCPs (−50 %, +65 %). Although the emission factors are based on local experiments, emissions from domestic fossil fuel and biomass burning still have considerable uncertainties (−38 %, +62 %) and (−38 %, +51 %), respectively.

## 4 Conclusions

This study introduces the newly mapped VOC and POA inventories (both traditional and full-volatility) for CRACMM and presents the first comprehensive evaluation of PM_2.5_ predictions using the newly developed CRACMM chemical mechanism. The performance of CRACMM with CB6r3_ae7, and Saprc07tic_ae7i are compared, and results demonstrate that CMAQ with CRACMM provides reliable predictions of PM_2.5_ and its components across China during the months of January, April, July, and October 2021, although there are discrepancies in some complex regions.

In conclusion, the comparison of the three chemical mechanisms using the traditional POA inventory reveals that differences in *R* and MB values are generally small. However, with the replacement of the full-volatility inventory, CRACMM tends to predict lower PM_2.5_ concentrations in January across most regions of China except PRD and YRD. In the other months, CRACMM predicts higher concentrations than CB6r3_ae7 and Saprc07tic_ae7i when the full-volatility inventory is incorporated. The differences in PM_2.5_ concentrations in January, are primarily attributed to lower POA concentrations, which are influenced by semi-volatile partitioning and reduced aging of semi-volatile POA species under lower temperatures. In contrast, CRACMM simulates elevated SOA concentrations in eastern China due to enhanced precursor availability, while reduced SOA formation is observed in the SCB, where winter conditions slow the oxidation of precursors. The inclusion of the full-volatility inventory in CRACMM results in higher SOA concentrations in October, driven by increased precursor availability. Overall, CRACMM demonstrates improved performance in terms of *R* and MB, particularly in January and October for the PRD region, but performs less well in April and July, particularly in the BTH region, compared to CB6r3_ae7. Additionally, CRACMM with the full-volatility inventory increase in simulated PM_2.5_ concentrations, resulting in smaller deviations from observation across many regions, highlighting the importance of including S/IVOC emissions in the chemical mechanism. Emission perturbation simulations using CMAQ further emphasize the significant role of various emission species, particularly BTX and SVOC, in driving PM_2.5_ formation. The SOA contribution from BTX emissions accounts for nearly 50 % of the PM_2.5_ changes, while S/IVOC emissions primarily influence PM_2.5_ through SOA formation. BTX emissions had a more significant impact in CB6r3_ae7, particularly in central China, partly due to the fewer VOC species included in the lumped BTX of the CRACMM mechanism. Future assessments of O_3_ predictions with CRACMM will offer additional constraints on the gas and aerosol chemistry that contributes to PM_2.5_ formation.

## Supplementary Material

Supplement1

*Supplement*. The supplement related to this article is available online at https://doi.org/10.5194/gmd-19-2531-2026-supplement.

## Figures and Tables

**Table 1.** Description of simulation scenarios and their emissions.

Scenarios	Mechanisms	POA emission inventory	Anthropogenic + Biogenic emission inventory
1	CB6r3_ae7	Traditional POA inventory	MEIC + MEGAN
2	Saprc07tic_ae7i	Traditional POA inventory	MEIC + MEGAN
3	CRACMM	Traditional POA inventory	MEIC + MEGAN
4	CRACMM	Full-volatility inventory	MEIC + MEGAN

**Table 2.** Monthly averaged metrics of PM_2.5_ evaluation and the number of monitoring sites.

Months	R	IOA	NMB	NME	No.
January	0.46	0.61	−19%	43%	1388
April	0.41	0.52	−29%	47%	1402
July	0.36	0.49	−32%	43%	1309
October	0.68	0.72	−12%	45%	1358
Recommend benchmark (Huang et al., 2021b)	> 0.60	> 0.70	< ±45%	< ±55%	–

**Table 3.** List of emission reductions relative to the base simulations in CMAQ-CRACMM.

Chemical Mechanism	Emission Reduction
CRACMM	Benzene, toluene and xylene-like emissions set to zero
CRACMM	Biogenic-ROC emissions set to zero
CRACMM	IVOC emissions set to zero
CRACMM	SVOC emissions set to zero
CRACMM	HC10 zero out (decane and species of similar reactivity)

## Data Availability

The model simulation is based on the CMAQ v5.4 developed by the US EPA, and the code is publicly available at https://doi.org/10.5281/zenodo.7218076 (US EPA Office of Research and Development, 2022). Biogenic emissions were estimated using the MEGANv3.2 model (Guenther et al., 2012), which is available at https://doi.org/10.5281/zenodo.10939297 (Guenther, 2024). All input data to reproduce the results and figures in this paper, has been archived on Zenodo (https://doi.org/10.5281/zenodo.18222704, Su and Chen, 2025) and is freely accessible.
