# Supplementary material for: Application and evaluation of CRACMM v1.0 mechanism in PM2.5 simulation over China
Source: Geosci Model Dev. Author manuscript; Available in PMC 2026 May 5. (PMC13137371; doi:10.5194/gmd-19-2531-2026)
Supplement: Supplement1 [file NIHMS2164974-supplement-Supplement1.pdf]

Supplement of Geosci. Model Dev., 19, 2531–2550, 2026
https://doi.org/10.5194/gmd-19-2531-2026-supplement
© Author(s) 2026. CC BY 4.0 License.

Supplement of

Application and evaluation of CRACMM v1.0 mechanism in $PM_{2.5}$ simulation over China

Qingfang Su et al.

Correspondence to: Yangjun Wang (yjwang326@shu.edu.cn) and Li Li (lily@shu.edu.cn)

The copyright of individual parts of the supplement might differ from the article licence.

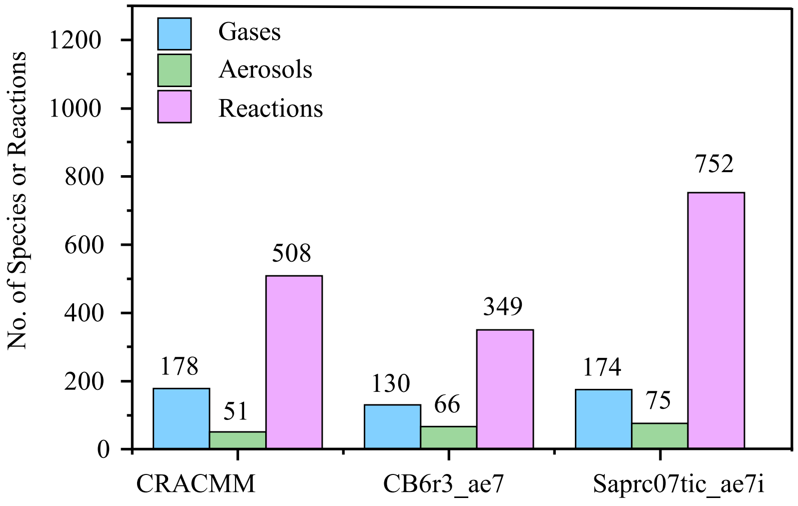

Figure S1. Number of chemical reactions, gas-phase species, and particle-phase species in three chemical mechanisms implemented in CMAQ.

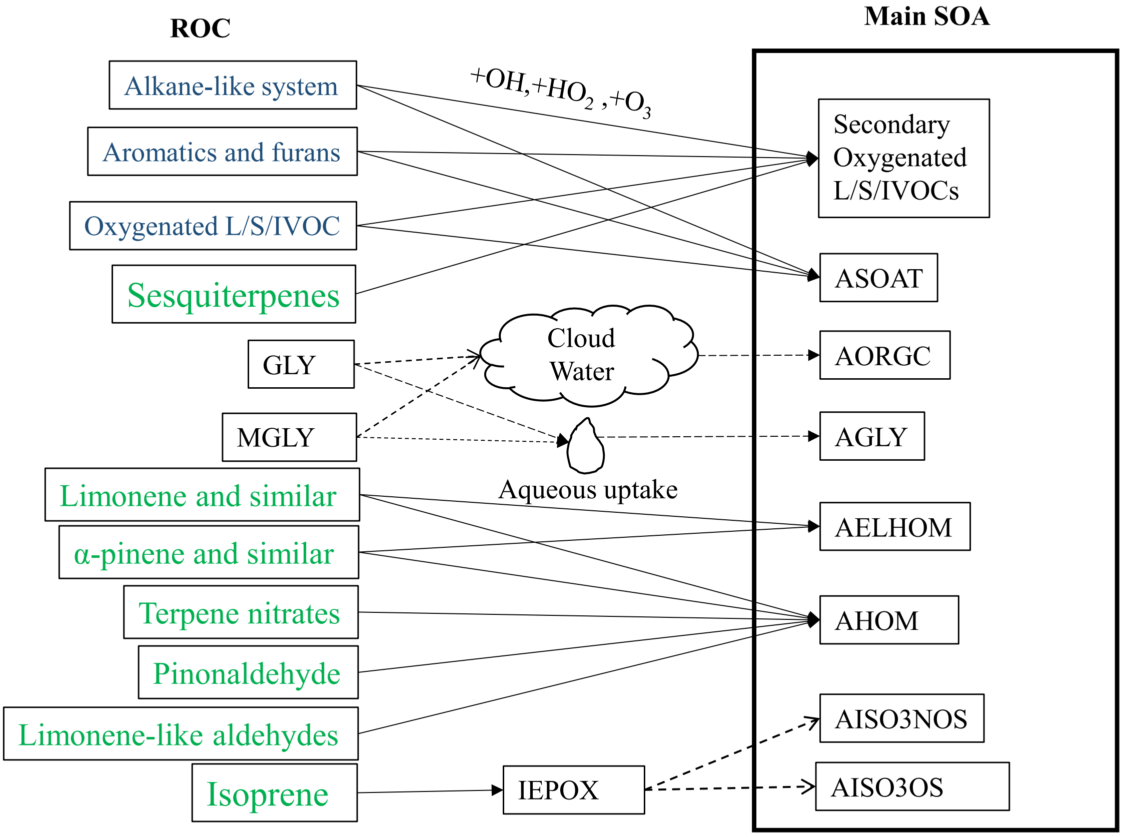

Figure S2. Treatment of SOA chemistry within the CRACMM mechanism in the CMAQv5.4 model. The thick black box surrounds all aerosol-phase species. Single-headed arrows represent irreversible processes. Dashed lines represent processes that modulated by the abundance of liquid water in the condensed phase. Species in dark blue are treated as anthropogenic species, and in green are treated as Biogenic species. The definitions of all surrogates are provided in Pye et al. (2023).

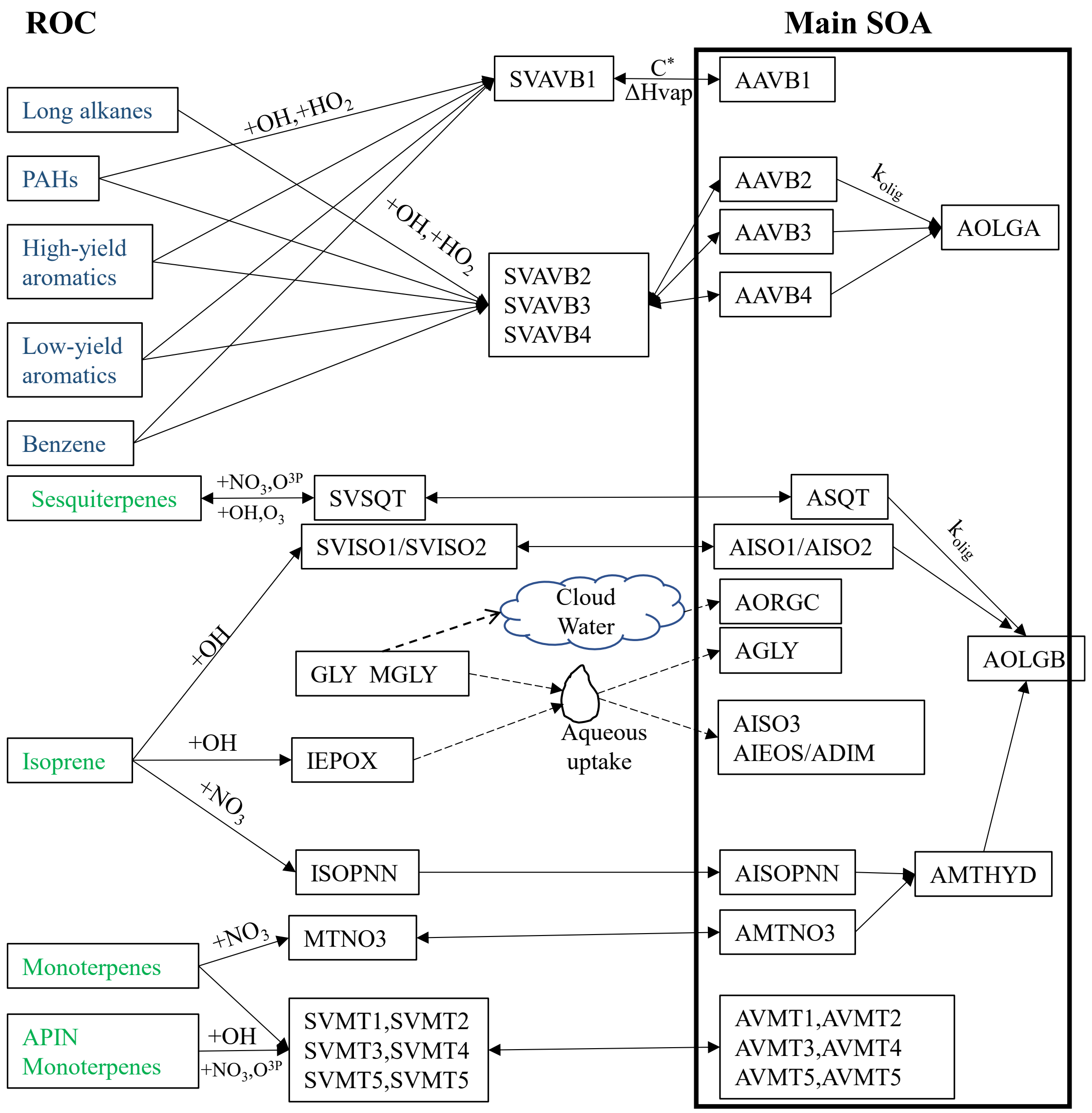

Figure S3. Treatment of SOA chemistry within the CB6 mechanism, including the mapping between chemical precursors and model species, in the CMAQv5.4 model in AERO7 (https://www.epa.gov/cmaq/how-cite-cmaq). The thick black box surrounds all aerosol-phase species. Double-headed arrows represent reversible processes, and single-headed arrows represent irreversible processes. Dashed lines represent processes that are dependent on the abundance of liquid water in the condensed phase. Species in dark blue are treated as anthropogenic species, and green are treated as biogenic species.

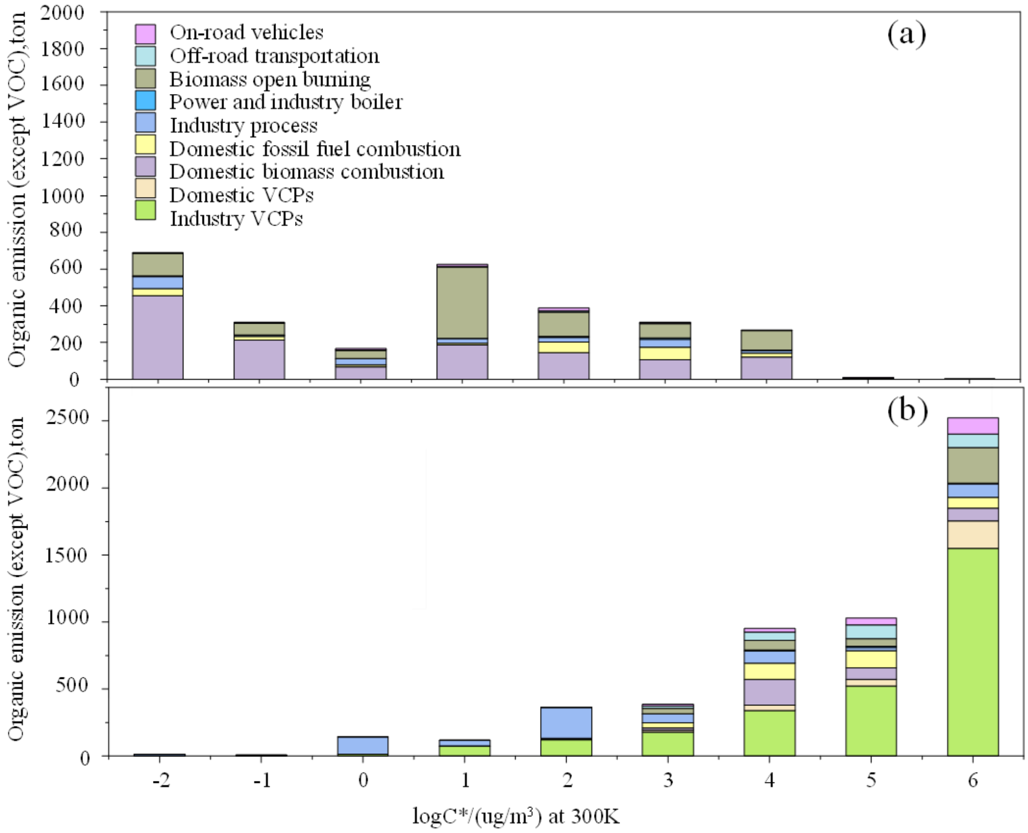

Figure S4. Anthropogenic L/S/IVOC emission inventory for China, binned by volatility: (a) particle-phase emissions, (b) gas-phase emissions.

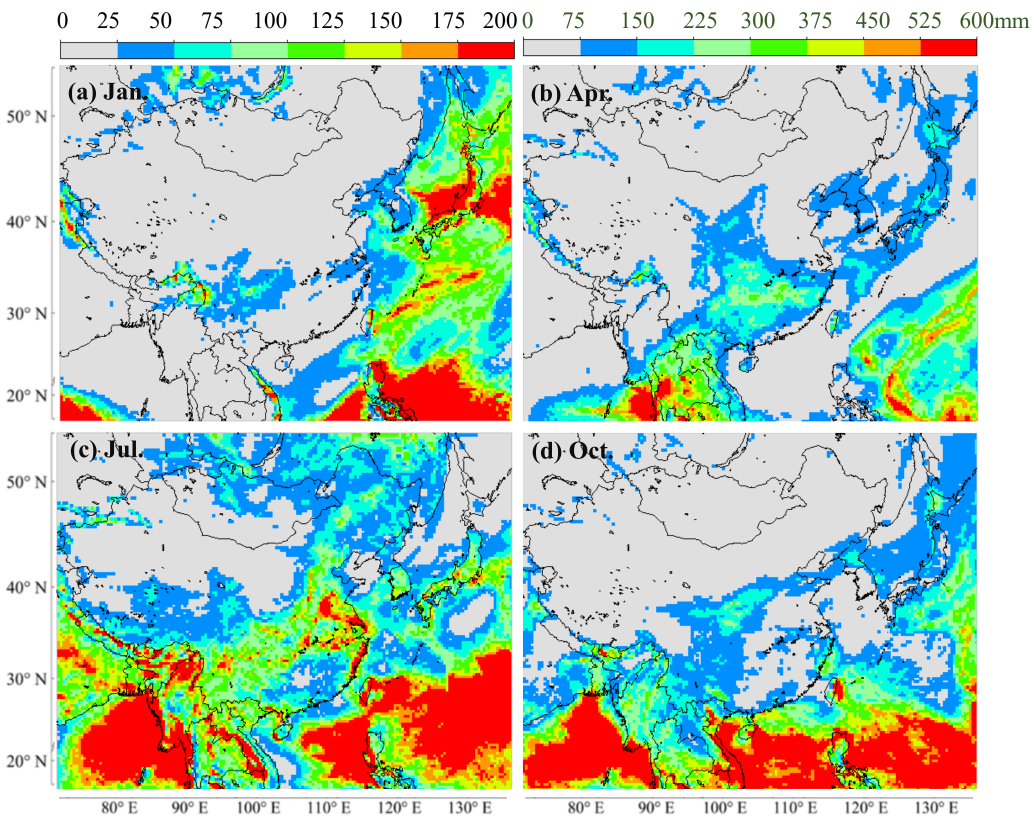

Figure S5. Spatial distribution of monthly total precipitation (mm). Note: Panels (a), (b), and (d) share a common color bar (top left), whereas panel (c) uses a separate color bar (top right) to better highlight spatial variations.

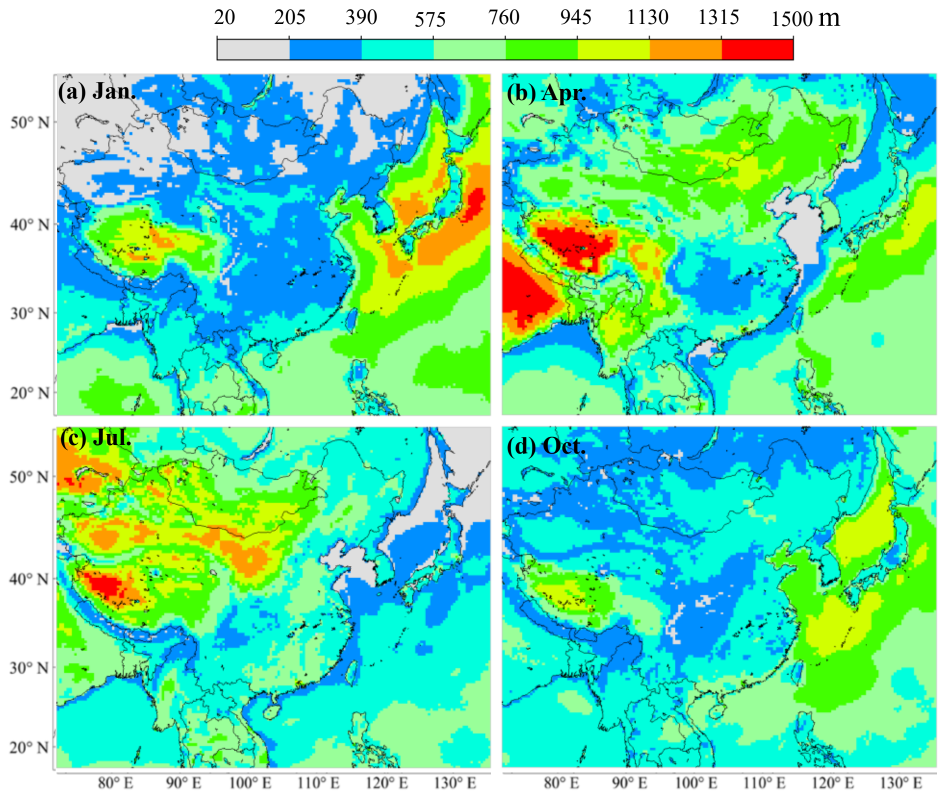

Figure S6. Spatial distribution of monthly mean PBL height (m).

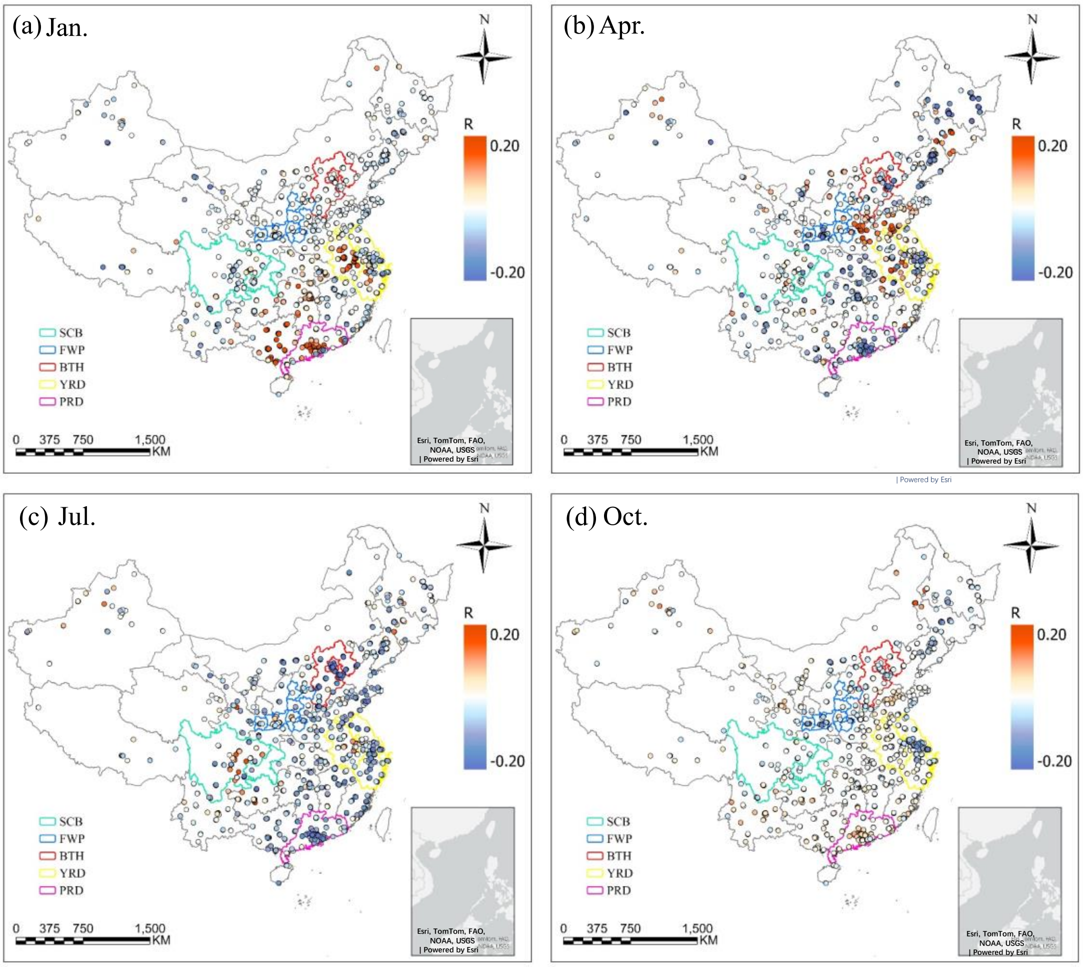

Figure S7. Differences in the R values of $PM_{2.5}$ between CRACMM（full-volatility inventory）and CB6r3_ae7, evaluated for January, April, July, and October of 2021 (|Powered by Esri).

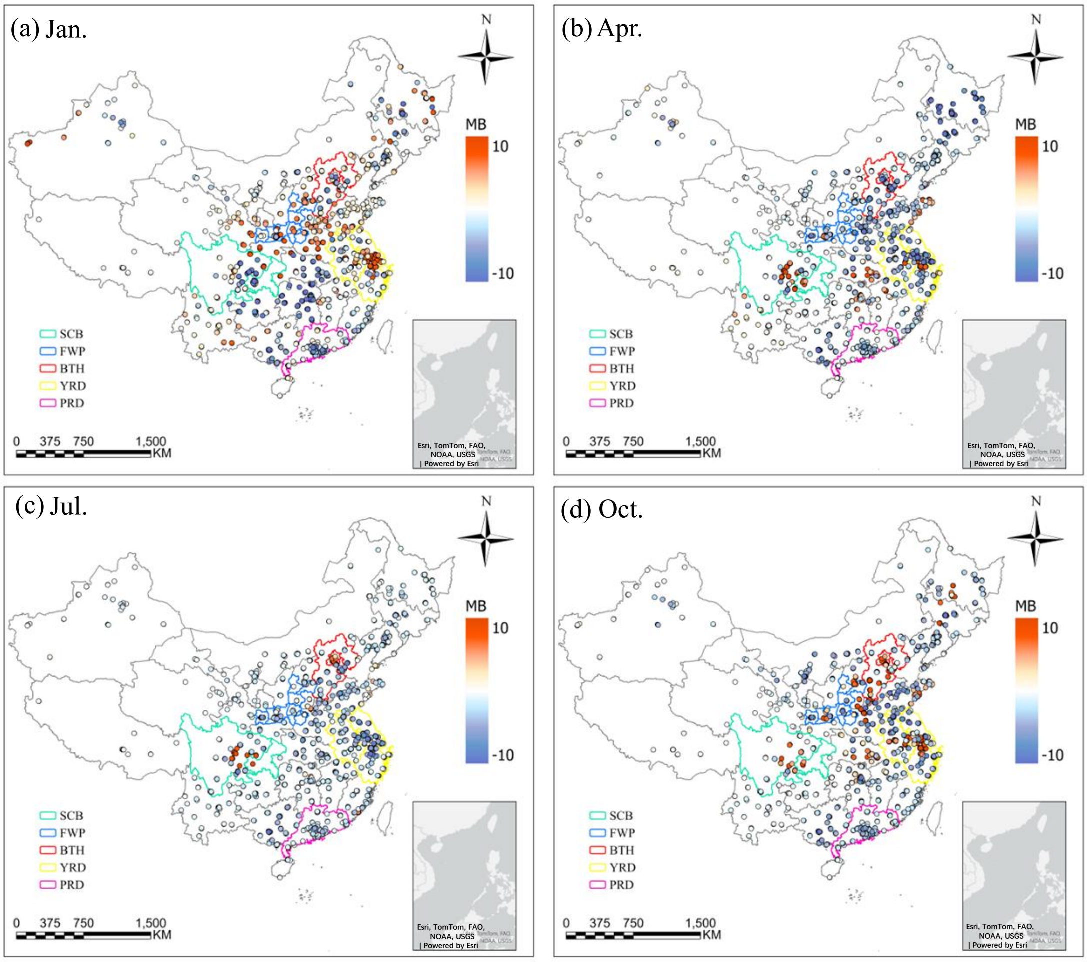

Figure S8. Differences in the MB values of $PM_{2.5}$ between CRACMM（full-volatility inventory） and CB6r3_ae7, evaluated for January, April, July, and October of 2021 (|Powered by Esri).

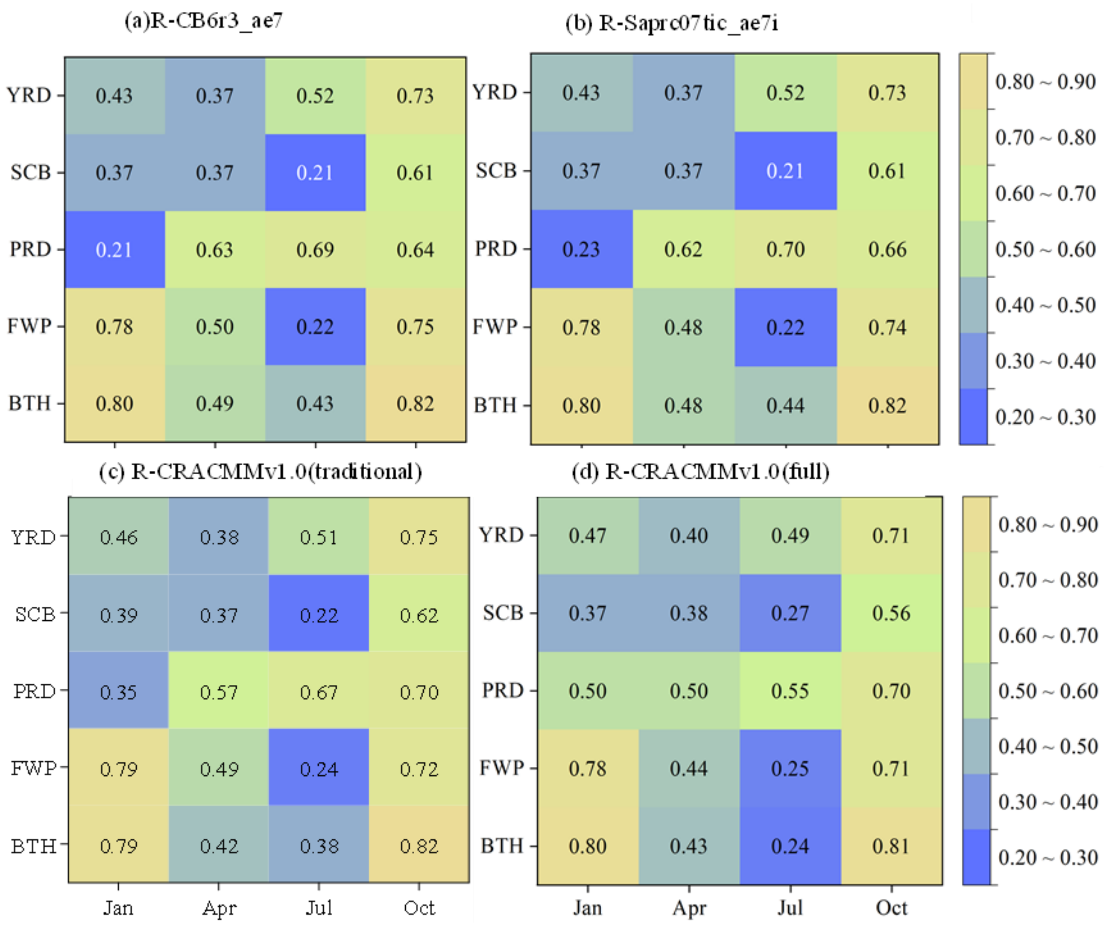

Figure S9. R values across five regions (YRD, SCB, PRD, FWP, BTH) and four months (January, April, July, October) for $PM_{2.5}$ estimated using (a) CB6r3_ae7, (b) Saprc07tic_ae7i, (c) CRACMM with traditional POA inventory and (d) CRACMM with full-volatility inventory.

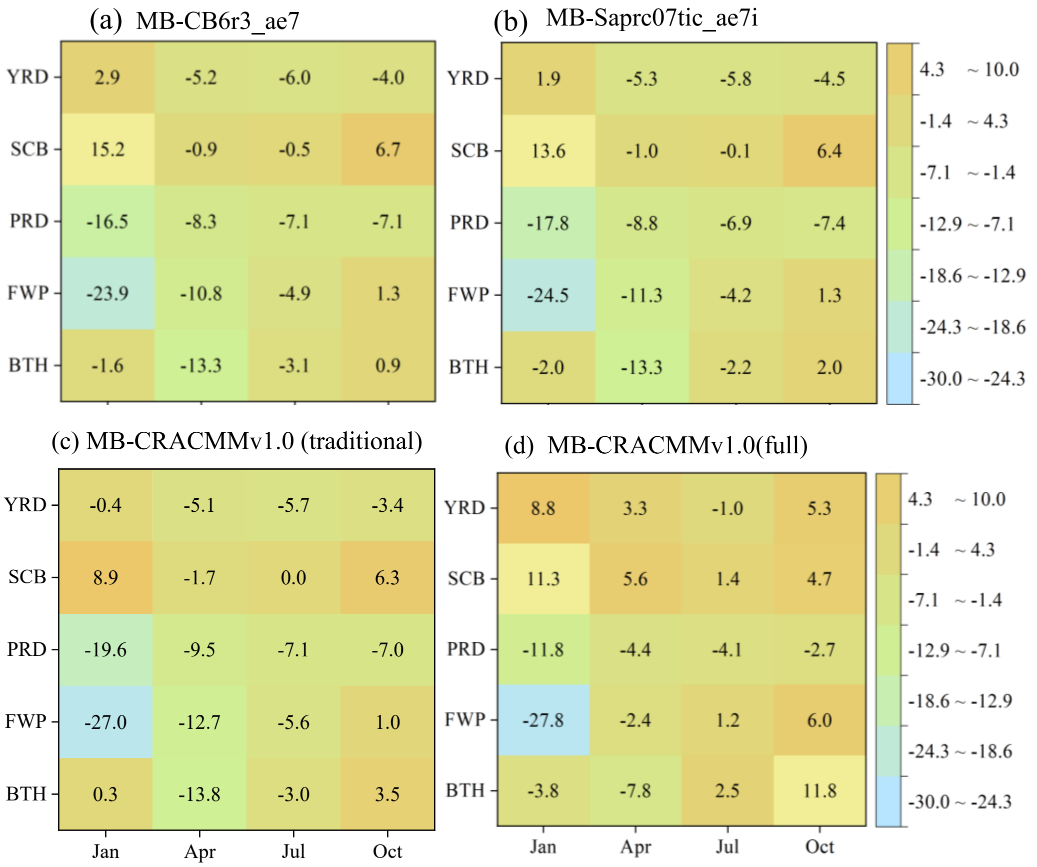

Figure S10. MB (μg/m³) across five regions (YRD, SCB, PRD, FWP, BTH) and four months (January, April, July, October) for $PM_{2.5}$ using (a) Cb6r3_ae7, (b) Saprc07tic_ae7i, (c) CRACMM with traditional POA inventory and (d) CRACMM with full-volatility inventory.

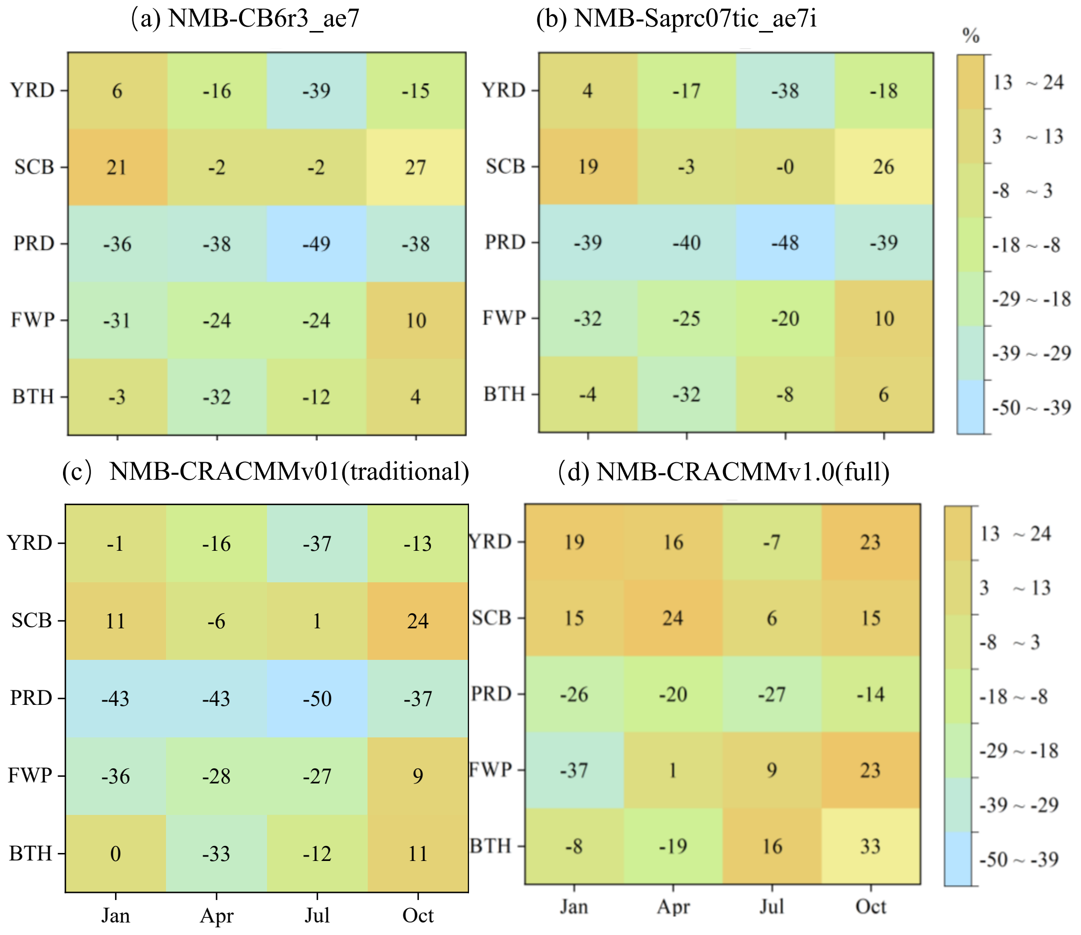

Figure S11. NMB (%) across five regions (YRD, SCB, PRD, FWP, BTH) and four months (January, April, July, and October) for (a) CB6r3_ae7, (b) Saprc07tic_ae7i, (c) CRACMM with traditional POA inventory, and (d) CRACMM with full-volatility POA inventory.

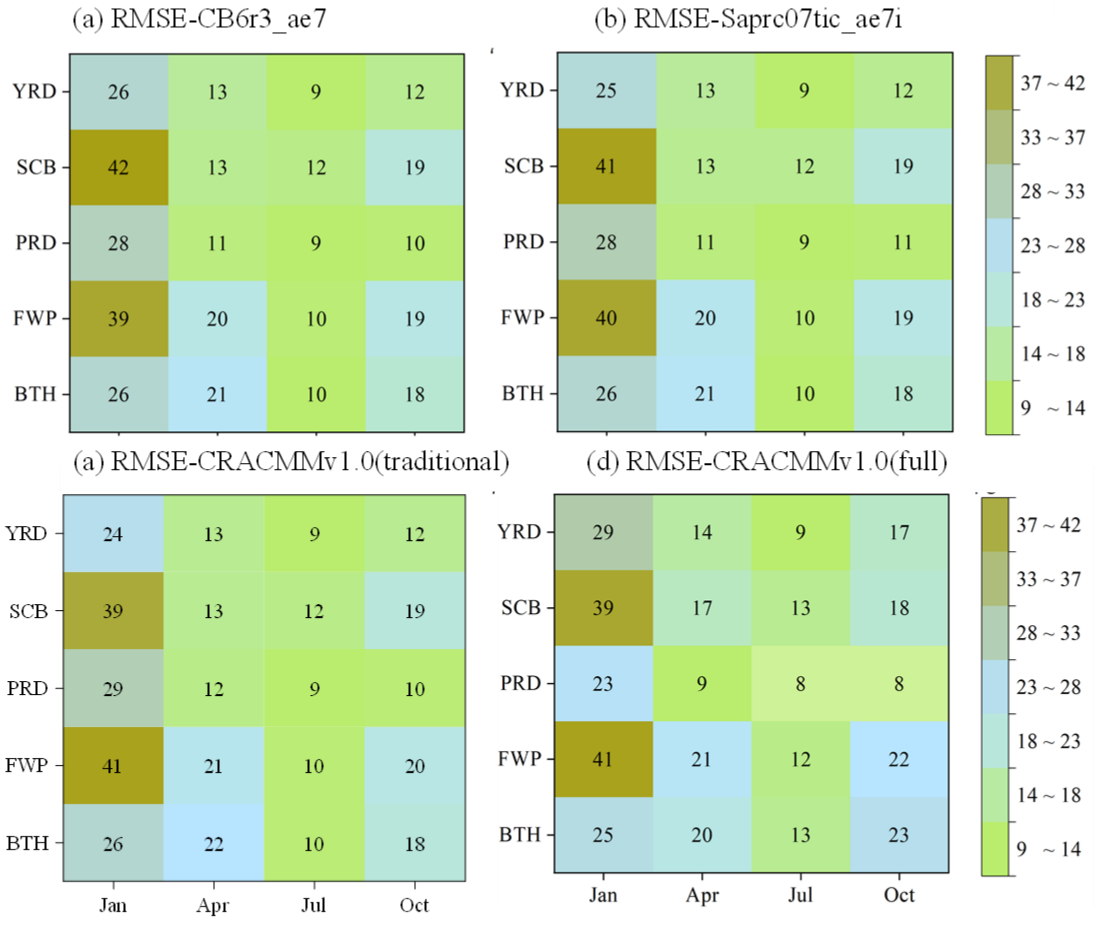

Figure S12. RMSE (μg/m^3) across five regions (YRD, SCB, PRD, FWP, BTH) and four months (January, April, July, and October) for (a) CB6r3_ae7, (b) Saprc07tic_ae7i, (c) CRACMM with traditional POA inventory, and (d) CRACMM with full-volatility POA inventory.

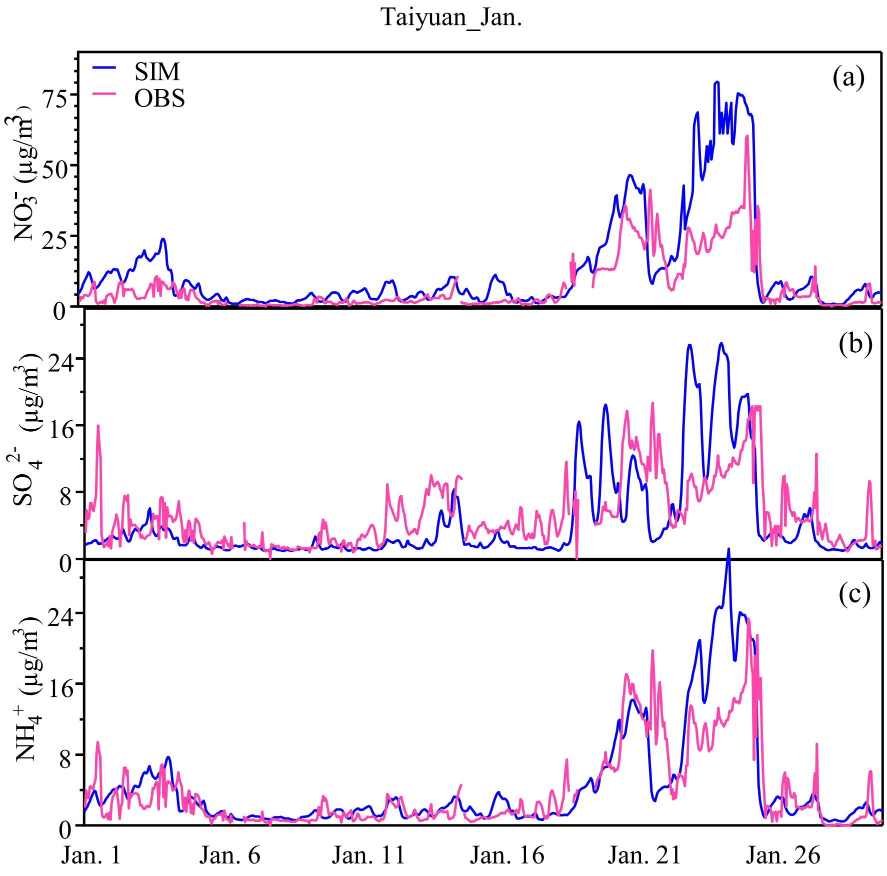

Figure S13. Comparison of hourly concentrations of (a) NO_3^-, (b) SO_4^{2-}, (c) NH_4^+ at Taiyuan in January 2021, based on ground-based observations (pink) and model simulations (blue).

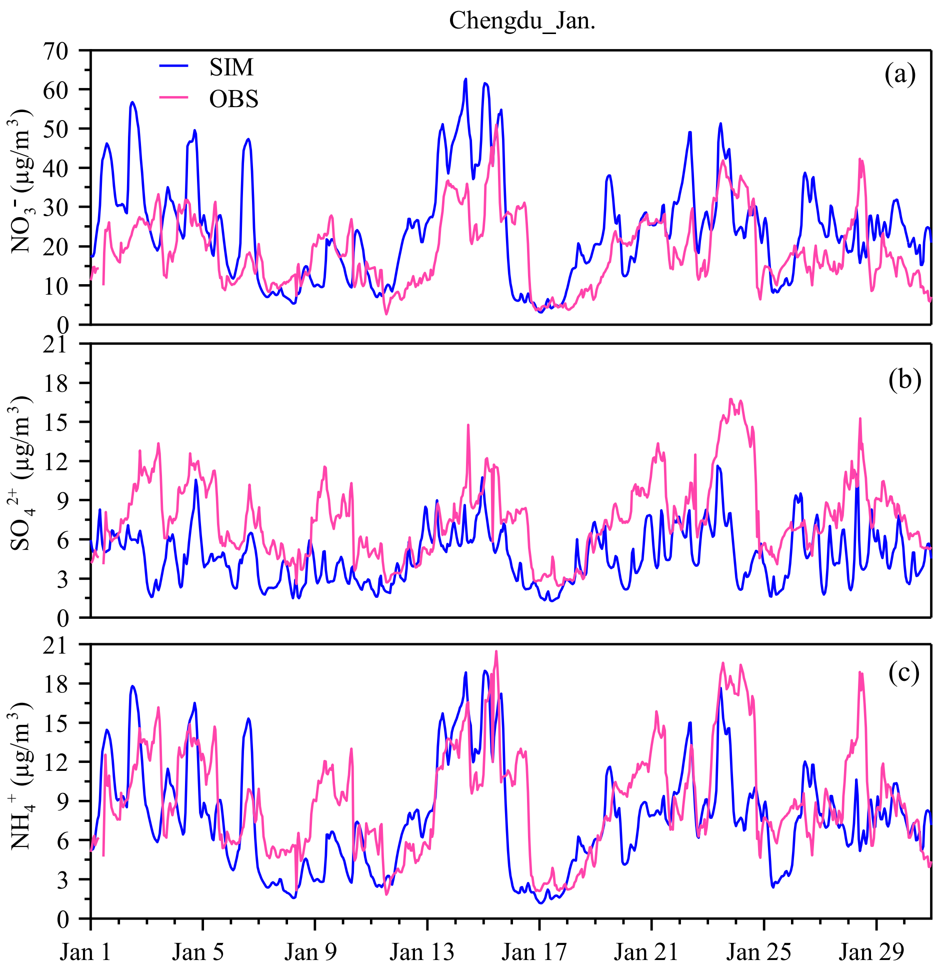

Figure S14. Comparison of hourly concentrations of (a) NO_3^-, (b) SO_4^{2-}, (c) NH_4^+ at Chengdu in January 2021, based on ground-based observations (pink) and model simulations (blue).

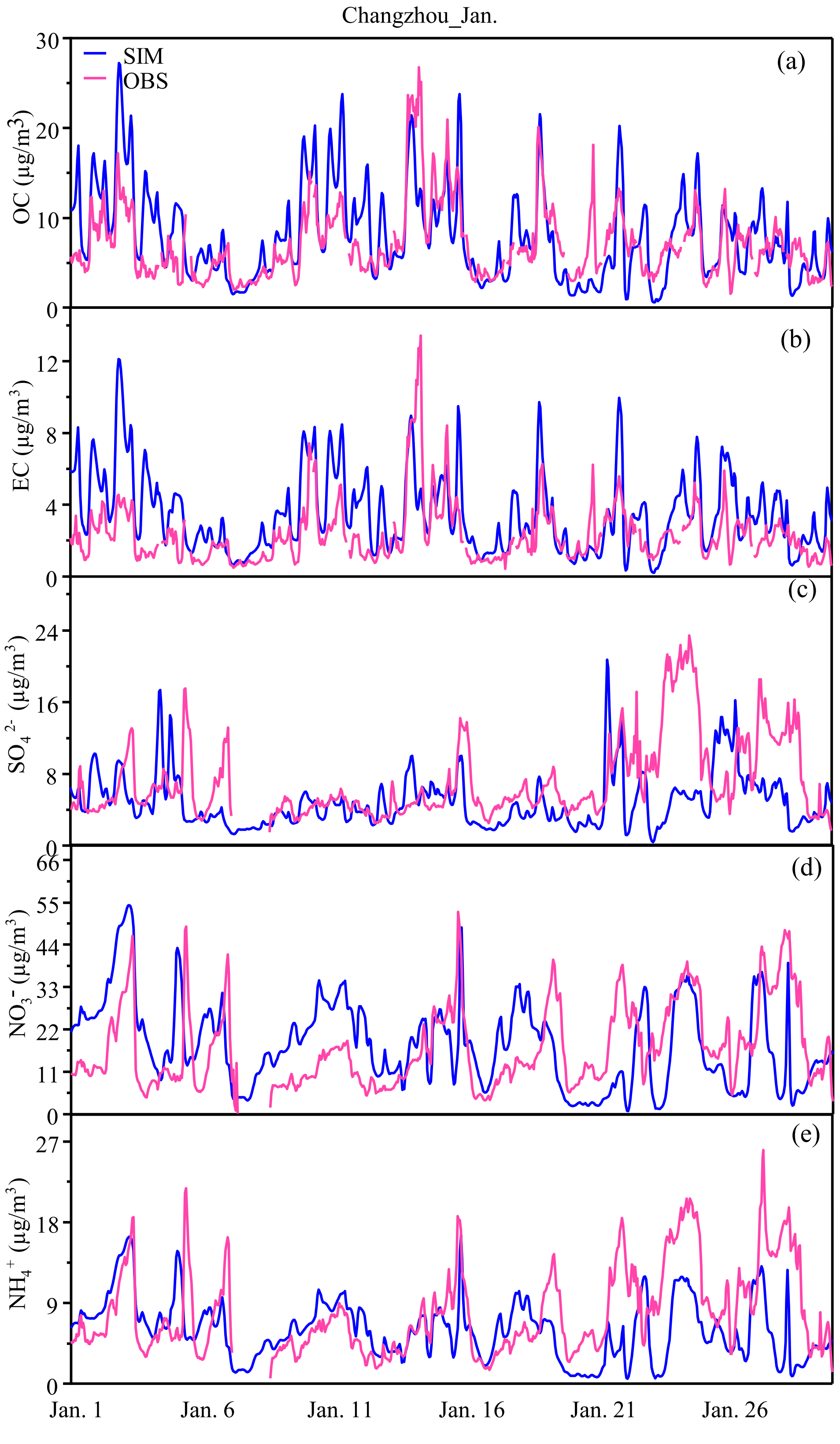

Figure S15. Comparison of hourly concentrations of (a) OC, (b) EC, (c) SO_4^{2-}, (d) NO_3^-, (e) NH_4^+ at Changzhou in January 2021, based on ground-based observations (pink) and model simulations (blue).

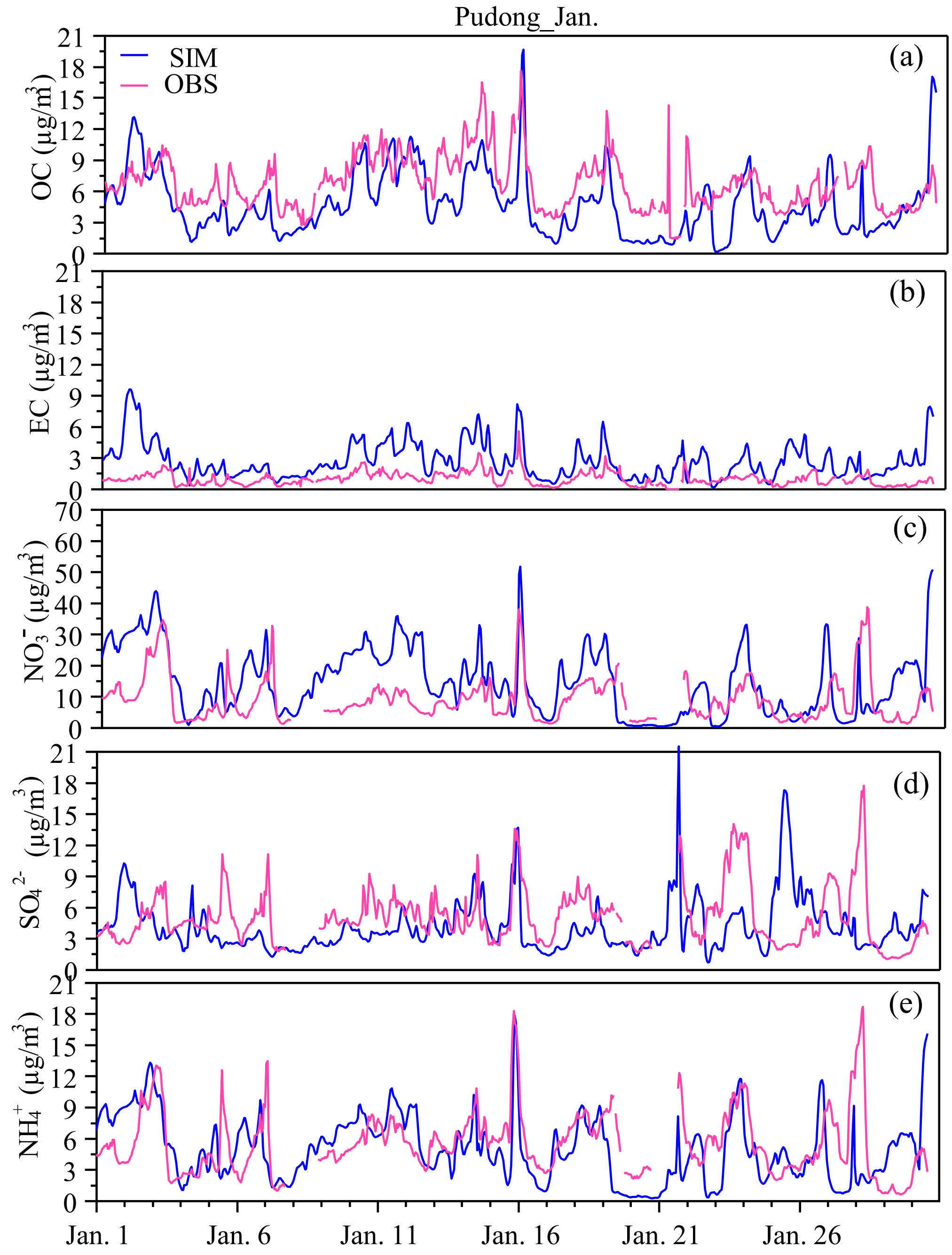

Figure S16. Comparison of hourly concentrations of (a) OC, (b) EC, (c) SO_4^{2-}, (d) NO_3^-, (e) NH_4^+ at Pudong in January 2021, based on ground-based observations (pink) and model simulations (blue).

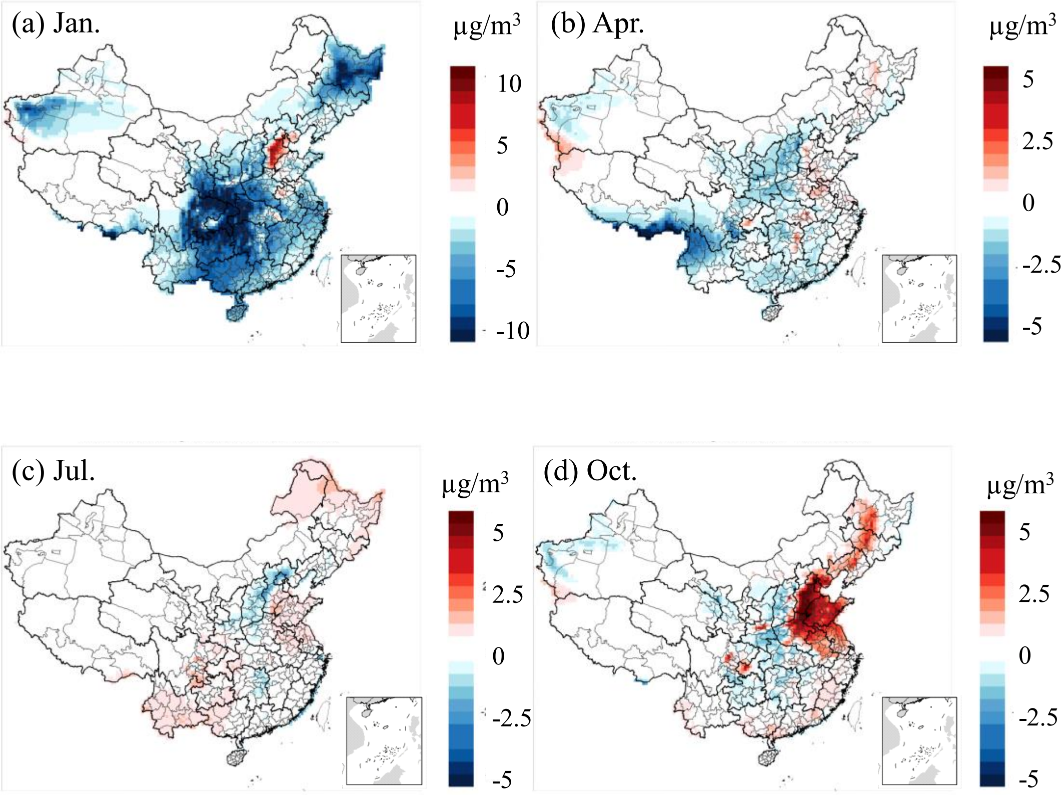

Figure S17. Differences in model-predicted $PM_{2.5}$ concentrations between CRACMM （traditional POA inventory) and CB6r3_ae7. Note that the color scale for panel (a) differs from panels (b–d) to highlight variations in the data.

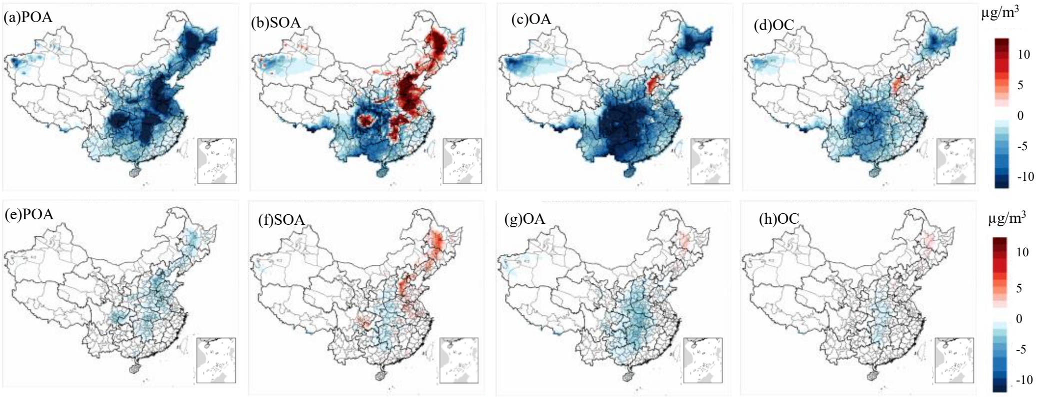

Figure S18. Differences in model-predicted $PM_{2.5}$ components—(a) POA, (b) SOA, (c) OA, and (d) OC for January, and (e) POA, (f) SOA, (g) OA, and (h) OC for October—between CRACMM (with the traditional POA inventory) and CB6r3_ae7. Figures (a–d) share the same scale, as do figures (e–h).

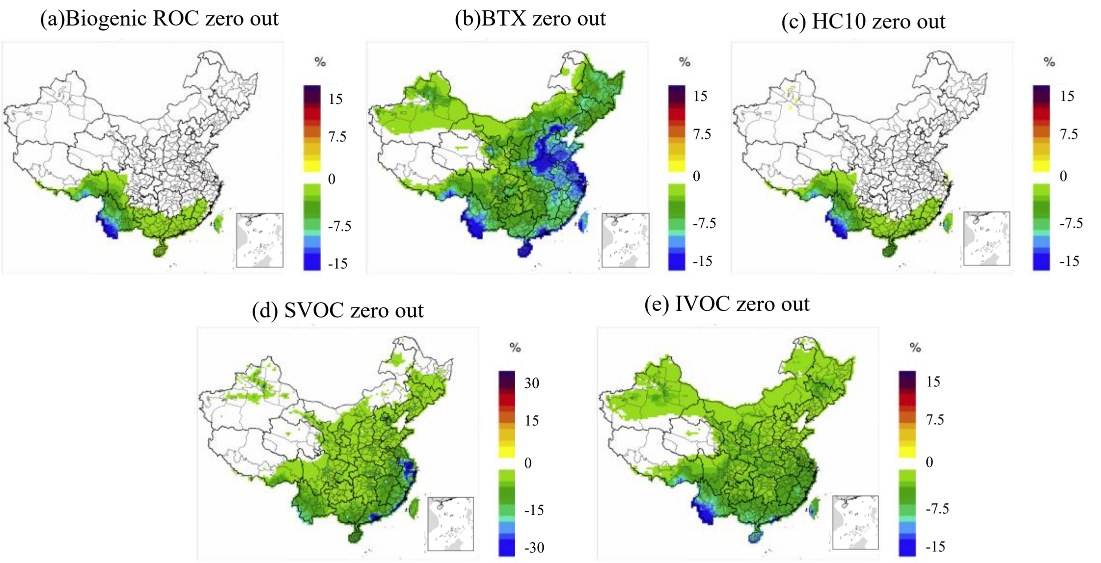

Figure S19. Percent changes in $PM_{2.5}$ concentrations between each zero-out scenario and its corresponding base simulation: (a)biogenic emissions, (b) BTX, (c) HC10, (d) SVOC, and (e) IVOC. Note that the color scale for panel (d) differs from other panels to highlight variations in the data.

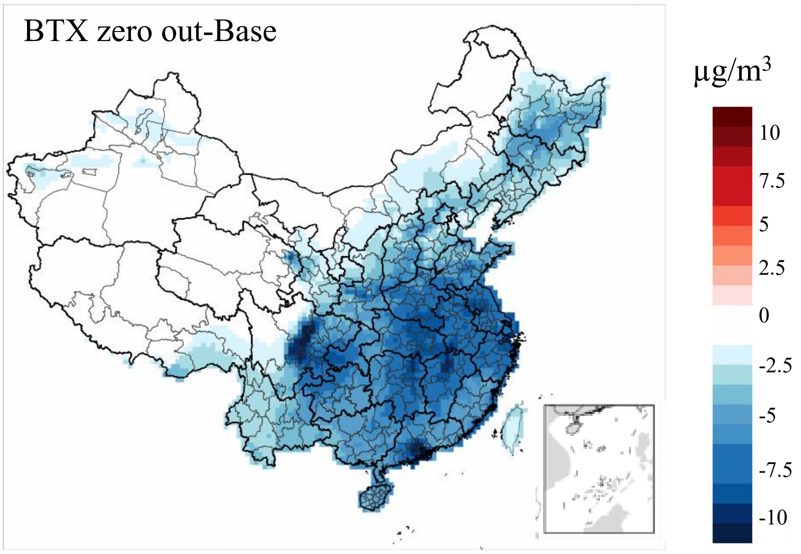

Figure S20. Difference in average O_3 concentrations between the BTX zero-out scenario and the base CRACMM simulation using the full-volatility inventory.

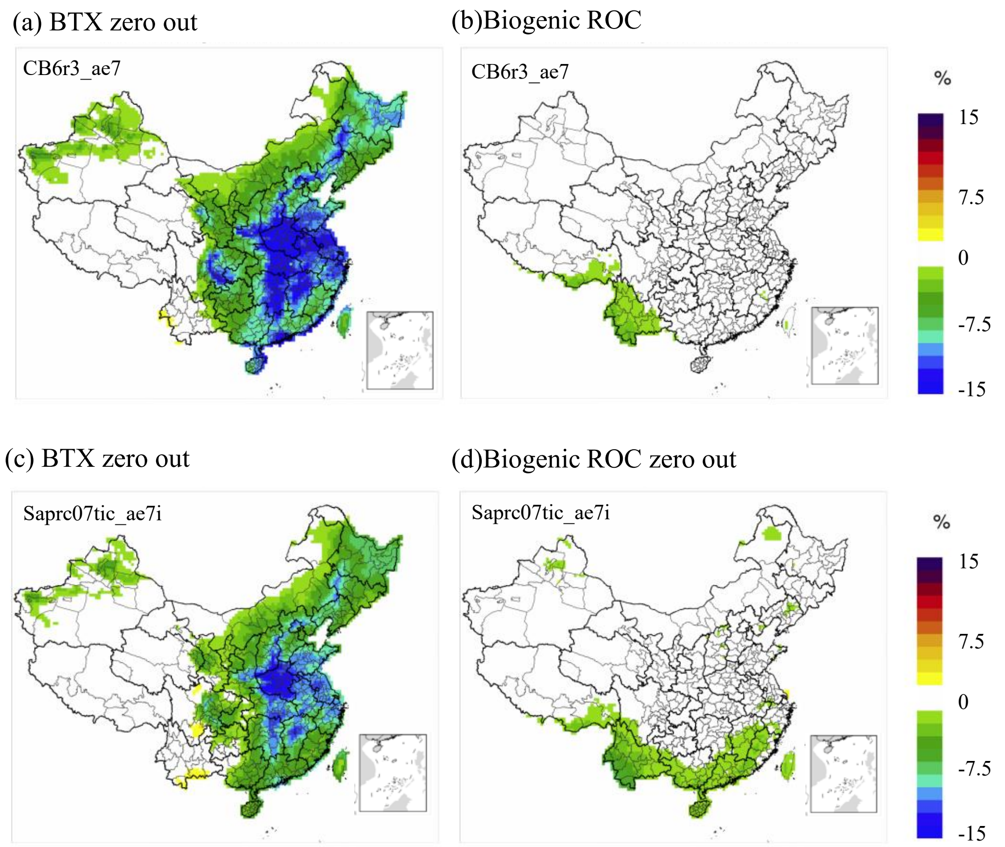

Figure S21. Percent changes in $PM_{2.5}$ concentrations between each zero-out scenario and its corresponding base simulation: (a) BTX, (b) biogenic ROC for CB6r3_ae7, (c) BTX, and (d) biogenic ROC for Saprc07tic_ae7i.

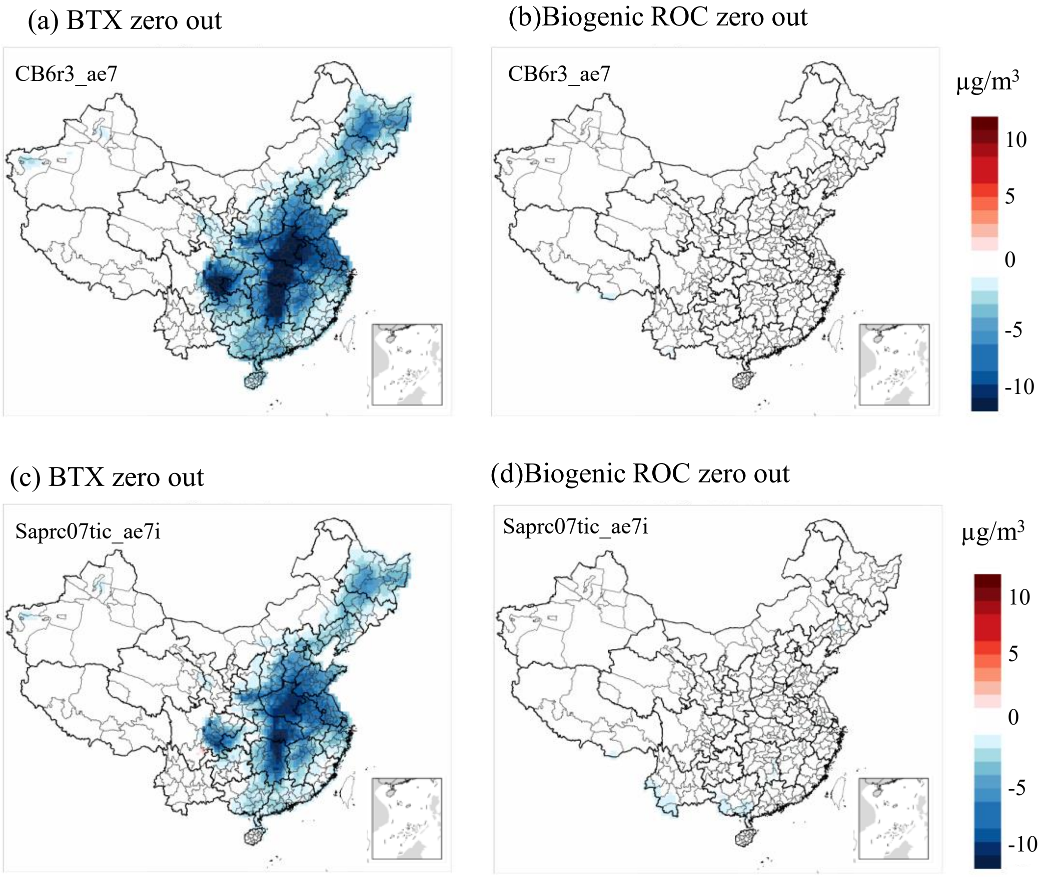

Figure S22. Changes in $PM_{2.5}$ concentrations between each zero-out scenario and its corresponding base simulation: (a) BTX and (b) biogenic ROC for CB6r3_ae7, and (c) BTX and (d) biogenic ROC for Saprc07tic_ae7i.

Table S1. Provincial VOC emissions in January 2019 from MEIC, categorized by sector. Units: 10^3 tons.

Regions	Power	Industry	Residential	Transportation	Agriculture
Beijing	0.01	2.54	0.62	0.69	0.00
Tianjin	0.01	2.47	0.34	0.39	0.00
Hebei	0.02	6.49	5.15	2.03	0.00
Shanxi	0.03	2.92	1.88	0.87	0.00
Inner Mongolia	0.05	3.03	2.55	0.74	0.00
Liaoning	0.02	5.35	2.33	1.02	0.00
Jilin	0.01	2.66	1.89	0.57	0.00
Heilongjiang	0.01	2.15	3.87	0.60	0.00
Shanghai	0.01	4.19	0.23	0.51	0.00
Jiangsu	0.06	11.95	2.71	2.50	0.00
Zhejiang	0.03	10.25	1.32	2.07	0.00
Anhui	0.02	4.20	3.76	1.16	0.00
Fujian	0.01	5.95	1.17	0.95	0.00
Jiangxi	0.01	3.35	1.28	0.76	0.00
Shandong	0.05	11.07	4.04	2.99	0.00
Henan	0.03	5.17	2.30	1.96	0.00
Hubei	0.01	5.06	5.34	1.08	0.00
Hunan	0.01	4.08	5.94	1.03	0.00
Guangdong	0.04	10.86	1.70	3.07	0.00
Guangxi	0.01	5.65	1.36	0.92	0.00
Hainan	0.00	0.83	0.25	0.20	0.00
Chongqing	0.01	2.46	2.05	0.55	0.00
Sichuan	0.00	5.86	5.71	1.51	0.00
Guizhou	0.02	1.50	7.48	0.61	0.00
Yunnan	0.00	3.34	2.47	1.09	0.00
Tibet	0.00	0.01	0.41	0.06	0.00
Shaanxi	0.02	2.91	2.07	0.84	0.00
Gansu	0.01	1.09	1.31	0.45	0.00
Qinghai	0.00	0.36	0.21	0.15	0.00
Ningxia	0.01	0.70	0.24	0.20	0.00
Xinjiang	0.03	2.21	1.79	0.59	0.00
Total	0.55	130.62	73.79	32.14	0.00

Table S2. Provincial VOC emissions in April 2019 from MEIC, categorized by sector. Units: 10^3 tons.

Regions	Power	Industry	Residential	Transportation	Agriculture
Beijing	0.01	2.25	0.27	0.70	0.00
Tianjin	0.01	2.86	0.12	0.41	0.00
Hebei	0.02	7.85	1.57	2.07	0.00
Shanxi	0.03	3.27	0.45	0.89	0.00
Inner Mongolia	0.04	3.57	1.02	0.74	0.00
Liaoning	0.01	6.08	1.03	1.05	0.00
Jilin	0.01	2.63	0.79	0.58	0.00
Heilongjiang	0.01	2.74	1.53	0.60	0.00
Shanghai	0.01	4.22	0.22	0.53	0.00
Jiangsu	0.05	12.04	1.45	2.62	0.00
Zhejiang	0.03	11.63	0.90	2.18	0.00
Anhui	0.02	4.32	1.14	1.21	0.00
Fujian	0.01	6.04	1.14	0.98	0.00
Jiangxi	0.01	3.92	0.75	0.80	0.00
Shandong	0.04	15.03	1.71	3.06	0.00
Henan	0.02	6.62	0.84	2.05	0.00
Hubei	0.01	6.12	1.54	1.13	0.00
Hunan	0.00	4.44	1.67	1.08	0.00
Guangdong	0.04	11.42	1.67	3.14	0.00
Guangxi	0.00	3.14	1.32	0.94	0.00
Hainan	0.00	0.70	0.25	0.20	0.00
Chongqing	0.00	2.97	0.61	0.57	0.00
Sichuan	0.00	6.59	1.60	1.55	0.00
Guizhou	0.02	1.53	1.89	0.64	0.00
Yunnan	0.00	3.15	1.28	1.11	0.00
Tibet	0.00	0.07	0.16	0.06	0.00
Shaanxi	0.01	3.39	0.64	0.86	0.00
Gansu	0.01	1.34	0.30	0.46	0.00
Qinghai	0.00	0.49	0.09	0.15	0.00
Ningxia	0.01	0.85	0.11	0.20	0.00
Xinjiang	0.02	2.76	0.72	0.60	0.00
Total	0.44	144.05	28.74	33.18	0.00

Table S3. Provincial VOC emissions in July 2019 from MEIC, categorized by sector. Units: 10^3 tons.

Regions	Power	Industry	Residential	Transportation	Agriculture
Beijing	0.01	2.32	0.27	0.70	0.00
Tianjin	0.01	2.68	0.12	0.41	0.00
Hebei	0.02	7.87	1.59	2.12	0.00
Shanxi	0.03	3.65	0.46	0.92	0.00
Inner Mongolia	0.05	3.56	0.59	0.78	0.00
Liaoning	0.02	6.46	0.67	1.07	0.00
Jilin	0.01	2.85	0.49	0.60	0.00
Heilongjiang	0.01	2.82	0.88	0.63	0.00
Shanghai	0.01	4.01	0.22	0.55	0.00
Jiangsu	0.06	11.74	1.46	2.62	0.00
Zhejiang	0.02	11.57	0.91	2.32	0.00
Anhui	0.02	4.41	1.17	1.27	0.00
Fujian	0.01	5.77	1.17	1.03	0.00
Jiangxi	0.01	4.00	0.77	0.84	0.00
Shandong	0.04	14.59	1.73	3.13	0.00
Henan	0.03	7.18	0.86	2.05	0.00
Hubei	0.01	5.97	1.58	1.13	0.00
Hunan	0.01	4.57	1.72	1.15	0.00
Guangdong	0.05	11.74	1.70	3.33	0.00
Guangxi	0.00	3.22	1.36	0.99	0.00
Hainan	0.00	0.59	0.25	0.21	0.00
Chongqing	0.00	2.73	0.62	0.57	0.00
Sichuan	0.00	6.66	1.64	1.59	0.00
Guizhou	0.01	1.61	1.95	0.64	0.00
Yunnan	0.00	2.76	1.32	1.11	0.00
Tibet	0.00	0.08	0.08	0.07	0.00
Shaanxi	0.01	3.38	0.65	0.88	0.00
Gansu	0.00	1.35	0.31	0.47	0.00
Qinghai	0.00	0.47	0.06	0.15	0.00
Ningxia	0.01	0.96	0.07	0.21	0.00
Xinjiang	0.03	2.95	0.42	0.61	0.00
Total	0.52	144.52	27.09	34.14	0.00

Table S4. Provincial VOC emissions in October 2019 from MEIC, categorized by sector. Units: 10^3 tons.

Regions	Power	Industry	Residential	Transportation	Agriculture
Beijing	0.01	2.47	0.27	0.69	0.00
Tianjin	0.01	3.04	0.12	0.40	0.00
Hebei	0.02	8.26	1.59	2.07	0.00
Shanxi	0.03	3.34	0.46	0.89	0.00
Inner Mongolia	0.05	4.36	1.05	0.74	0.00
Liaoning	0.01	5.67	1.05	1.05	0.00
Jilin	0.01	2.93	0.81	0.57	0.00
Heilongjiang	0.01	3.12	1.57	0.60	0.00
Shanghai	0.01	4.64	0.22	0.53	0.00
Jiangsu	0.05	11.59	1.46	2.62	0.00
Zhejiang	0.02	12.46	0.91	2.18	0.00
Anhui	0.02	5.13	1.17	1.21	0.00
Fujian	0.01	6.68	1.17	0.98	0.00
Jiangxi	0.01	4.67	0.77	0.80	0.00
Shandong	0.04	14.22	1.73	3.13	0.00
Henan	0.02	7.51	0.86	2.05	0.00
Hubei	0.01	5.44	1.58	1.13	0.00
Hunan	0.01	5.38	1.72	1.08	0.00
Guangdong	0.04	13.84	1.70	3.14	0.00
Guangxi	0.01	3.55	1.36	0.94	0.00
Hainan	0.00	0.81	0.25	0.20	0.00
Chongqing	0.00	3.01	0.62	0.57	0.00
Sichuan	0.00	7.29	1.64	1.55	0.00
Guizhou	0.02	1.83	1.95	0.64	0.00
Yunnan	0.00	3.12	1.32	1.11	0.00
Tibet	0.00	0.05	0.16	0.06	0.00
Shaanxi	0.01	4.19	0.65	0.86	0.00
Gansu	0.01	1.57	0.31	0.46	0.00
Qinghai	0.00	0.49	0.10	0.15	0.00
Ningxia	0.01	1.11	0.07	0.20	0.00
Xinjiang	0.03	3.75	0.74	0.60	0.00
Total	0.45	155.52	29.37	33.21	0.00

Table S5. The species mapping from Sarprc07tic_ae7i and CB6r3_ae7 to CRACMM used in CMAQ, where "E" stands for explicit species and "L" stands for lumped species.

Saprc07/CB06	CRACMM	E/L	Saprc07/CB06	CRACMM	E/L
ACYE	ACE	E	ALDX	ALD	E
ACET	ACT	E	ETHA	ETH	E
CH4	ECH4	E	ETH	ETE	E
ETOH	EOH	E	MGLY	MGLY	L
HCHO	HCHO	E	GLY+CCHO*0.25	GLY	L
ETOH	EOH	E	MEK*0.001	HKET	L
HCHO	HCHO	E	CCHO*0.75	ACD	E
ISOP	ISO	E	TERP*0.65	LIM	L
MEOH	MOH	E	TERP*0.35	API	L
FACD	ORA1	E	ALK4*0.1	ROH	L
ACRO	ACRO	E	ALK5*0.03	PROG	L
ETOH	EOH	E	ALK4*0.9	HC5	L
BENZ	BEN	E	ALK5*0.97	HC10	L
MVK	MVK	L	MEK*0.75	MEK	L
ETH	ALK1	L	XYM*0.3	XYE	L
RNO3	ONIT	L	XYM*0.7	XYM	L
OLE1	OLI	L	MEK*0.25+PRD2	KET	L
OLE2	OLT	L	AACD+PACD	ORA2	L
CRES*0.25	CSL	L	ALK2+ALK3	HC3	L
CRES*0.75	PHEN	L	MACR+IPRD	MACR	L

Table S6. Emissions of VOCs by mechanism for January, April, July, and October. Units are in 10^4 tons/yr.

Mechanisms	January	April	July	October
Cb6r3_ae7	227.1	202.4	203.1	215.8
Saprc07tic_ae7i	237.6	211.3	211.1	220.0
CRACMM	221.1	205.1	202	212.8

Table S7. Properties of semi-volatile POA species as defined in CB6r3_ae7, Saprc07tic_ae7i, and CRACMM.

Species name	MW	C^*	O:C	Hvap	Mechanisms
ALVPO1/VLVPO1	218	0.1	0.185	89	
ASVPO1/VSVPO1	230	1	0.123	85	
ASVPO2/VSVPO2	241	10	0.073	81	
ASVPO3/VSVPO3	253	100	0.032	77	
AIVPO1/VIVPO1	266	1000	0	73	CB6r3_ae7/
ALVOO1/VLVOO1	136	0.01	0.886	93	Saprc07tic_ae7i
ALVOO2/VLVOO2	136	0.1	0.711	89	
ASVOO1/VSVOO1	135	1	0.567	85	
ASVOO2/VSVOO2	135	10	0.447	81	
ASVOO3/VSVOO3	134	100	0.345	77	
VROCN2OXY2/AROCN2OXY2	282	0.01	0.2	93	
VROCP0OXY2/AROCP0OXY2	242	1	0.2	85	
VROCP1OXY1/AROCP1OXY1	270	10	0.1	81	
VROCP2OXY2/AROCP2OXY2	200	100	0.2	77	
VROCP3OXY2/VROCP3OXY2	186	1000	0.2	73	
AROCN2ALK	422	0.01	0	104	CRACMM
AROCN1ALK	408	0.1	0	96	
AROCP0ALK	394	1	0	85	
AROCP1ALK	380	10	0	81	
AROCP2ALK	338	100	0	77	
AROCP3ALK	296	1000	0	73	

A: aerosol; **V**: vapor; **LV**: low volatility; **SV**: semi-volatile; **OO**: oxidized organic; **ROC**: reactive organic carbon; **OXY**: oxygen; **ALK**: alkene; **P**: positive; **N**: negative; **MW**: molecular weight ($g \cdot mol^{-1}$); **Hvap**: enthalpy of vaporization ($kJ \cdot mol^{-1}$).

Table S8. Mapping rules between 2D-VBS species and CRACMM L/S/IVOC species in the gas phase.

2D-VBS	CRACMM
CSM1O2C00P	VROCN1ALK
CS00O2C00P	VROCP0ALK
CS01O2C00P	VROCP1ALK
CS02O2C00P	VROCP2ALK
CS03O2C00P	VROCP3ALK
CS04O2C00P	VROCP4ALK
CS05O2C00P	VROCP5ALK
CS06O2C00P	VROCP6ALK
CS06O2C01P	VROCP6OXY1
CS05O2C01P	VROCP5OXY1
CS04O2C01P	
CS03O2C01P	
CS02O2C01P	
CS01O2C01P	VROCP1OXY1
CS00O2C01P	
CSM1O2C01P	
CSM1O2C02P	VROCN2OXY2
CS00O2C02P	VROCP0OXY2
CS01O2C02P	VROCP2OXY2
CS02O2C02P	
CS03O2C02P	VROCP3OXY2
CS04O2C02P	VROCP4OXY2
CS06O2C04P	
CS05O2C04P	
CS04O2C04P	VROCP0OXY4
CS03O2C04P	
CS02O2C04P	
CS01O2C04P	
CS00O2C04P	VROCN2OXY4
CSM1O2C04P	

Table S9. Locations of monitoring sites with observations of $PM_{2.5}$ components in each region.

Site	Region	Longitude (°)	Latitude (°)
Chengdu	SCB	104.079	30.636
Changzhou	YRD	119.891	38.011
Taiyuan	FWP	112.434	38.011
Pudong	YRD	121.533	31.228
Chongming	YRD	121.972	31.524
Dianshanhu	YRD	120.978	31.094

Table S10. Definition of model performance evaluation metrics used in this study.

Statistical metrics	Definition	Units
MB	$MB = \frac{1}{N}\sum_{i=1}^{N}(C_m - C_o)$	μg/m^3
NMB	$NMB = \frac{\sum_{i=1}^{N}(C_m - C_o)}{\sum_{i=1}^{N} C_o} \times 100\%$	%
NME	$NME = \frac{\sum_{i=1}^{N}\lvert C_m - C_o\rvert}{\sum_{i=1}^{N} C_o} \times 100\%$	%
RMSE	$RMSE = \sqrt{\frac{1}{N}\sum_{i=1}^{N}(C_m - C_0)^2}$	μg/m^3
R	$R = \frac{\sum_{i=1}^{N}(C_m - \overline{C_m})(C_o - \overline{C_o})}{\sqrt{\sum_{i=1}^{N}(C_m - \overline{C_m})}\sqrt{\sum_{i=1}^{N}(C_o - \overline{C_o})}}$	/
IOA	$IOA = 1 - \frac{\sum_{i=1}^{N}(C_m - C_o)^2}{\sum_{i=1}^{N}(\lvert C_m - \overline{C_0}\rvert + \lvert C_0 - \overline{C_0}\rvert)^2}$	/

Table S11. Evaluation of WRF-simulated temperature over five regions.

Month	Region	R	NMB	MB	RMSE	OBS-ave	SIM-ave
January	BTH	0.9	27.8	-2.0	2.9	-3.9	-5.9
	FWP	0.9	-13.5	-1.2	2.3	0.2	-1.0
	PRD	0.9	-4.6	-0.7	2.1	14.3	13.7
	SCB	0.9	11.3	-0.8	2.3	5.7	5.0
	YRD	1.0	-41.3	-1.8	2.4	4.6	2.9
April	BTH	0.8	-4.9	0.5	2.1	14.1	13.5
	FWP	0.9	-0.1	0.0	2.0	13.6	13.6
	PRD	0.7	6.6	1.6	2.5	23.9	25.5
	SCB	0.8	-3.7	-0.2	2.7	16.6	16.5
	YRD	0.8	0.3	-0.1	1.9	16.1	16.0
July	BTH	0.7	5.8	1.6	3.4	26.4	28.0
	FWP	0.7	8.2	2.2	4.3	26.3	28.4
	PRD	0.7	5.0	1.5	3.0	29.6	31.1
	SCB	0.5	-1.2	-0.1	3.8	25.9	25.8
	YRD	0.6	-1.5	-0.5	3.0	28.2	27.6
October	BTH	0.8	7.2	0.8	2.5	12.1	12.9
	FWP	0.5	10.6	1.2	4.9	11.2	12.4
	PRD	0.3	18.6	3.9	8.6	21.2	25.1
	SCB	0.6	1.1	0.4	5.2	14.7	15.0
	YRD	0.9	-0.9	-0.3	2.0	19.1	18.8

Table S12. Evaluation of WRF-simulated relative humidity over five regions.

Month	Region	R	NMB	MB	RMSE	OBS-ave	SIM-ave
January	BTH	0.8	-10.0	-4.3	12.3	44.4	40.1
	FWP	0.6	-3.6	-2.0	12.9	37.0	35.0
	PRD	0.7	-25.3	-14.4	19.1	55.5	41.2
	SCB	0.4	17.0	-7.8	26.0	65.1	57.3
	YRD	0.9	-10.2	-6.6	12.7	63.6	57.0
April	BTH	0.8	-18.9	-9.0	19.9	45.4	36.3
	FWP	0.9	-20.7	-13.0	17.4	60.2	47.2
	PRD	0.5	-16.9	-13.0	16.6	76.5	63.5
	SCB	0.6	-4.6	-5.8	15.5	72.6	66.9
	YRD	0.7	-12.1	-8.8	16.4	72.3	63.5
July	BTH	0.7	-16.8	-13.1	19.2	78.5	65.4
	FWP	0.5	-27.7	-19.7	26.6	69.9	50.2
	PRD	0.7	-12.7	-9.9	16.0	77.0	67.1
	SCB	0.4	-4.3	-3.7	17.9	77.5	73.8
	YRD	0.7	-1.3	-1.2	11.6	81.3	80.0
October	BTH	0.7	-18.7	-12.6	20.9	62.8	50.3
	FWP	0.2	-8.8	-6.2	27.6	68.4	62.3
	PRD	0.3	-6.8	-4.8	25.2	69.4	64.6
	SCB	0.3	8.5	5.2	30.5	73.8	79.0
	YRD	0.7	-6.9	-5.3	10.3	76.7	71.4

Table S13. Evaluation of WRF-simulated wind speed over five regions.

Month	Region	R	NMB	MB	RMSE	OBS-ave	SIM-ave
January	BTH	0.8	43.6	1.0	1.6	2.6	3.6
	FWP	0.6	20.1	0.3	1.1	2.6	2.9
	PRD	0.8	61.0	1.5	1.8	2.6	4.1
	SCB	0.4	178.7	0.9	1.3	1.6	2.5
	YRD	0.7	40.5	0.8	1.4	3.0	3.8
April	BTH	0.6	12.5	1.8	7.3	2.3	4.1
	FWP	0.6	21.9	0.4	1.4	3.1	3.5
	PRD	0.3	46.1	0.9	1.4	2.4	3.3
	SCB	0.5	50.1	0.8	1.3	2.1	2.9
	YRD	0.6	40.2	0.8	1.5	3.1	3.9
July	BTH	0.2	-34.0	5.9	145.4	-2.9	3.1
	FWP	0.2	14.8	0.2	2.2	2.6	2.8
	PRD	0.4	15.9	0.4	1.9	2.8	3.2
	SCB	0.1	-0.8	-0.1	1.6	1.9	1.9
	YRD	0.5	34.3	0.9	2.6	3.8	4.7
October	BTH	0.7	62.4	1.0	1.5	2.0	3.0
	FWP	0.3	28.9	0.3	1.3	2.0	2.3
	PRD	0.3	65.1	1.8	2.8	3.1	4.9
	SCB	0.2	58.9	0.7	1.4	1.5	2.2
	YRD	0.8	47.1	1.0	1.6	2.9	3.8

Table S14. Evaluation of $PM_{2.5}$ components simulations using CRACMM in selected cities during January 2021.

Month	Region	$PM_{2.5}$ components	NMB	MB	RMSE	NME	IOA	OBS	SIM
Jan.	Chengdu	NO_3^-	29.5	5.5	12.4	52.8	0.7	18.8	24.3
		SO_4^{2-}	-39.4	-3	4.2	42.1	0.6	7.7	4.6
		NH_4^+	-15.2	-1.4	4.1	35.4	0.7	9.1	7.7
	Chongming	OC	14.7	0.6	2.6	44.0	0.7	4.2	4.8
		EC	212.0	1.9	2.5	215.8	0.3	0.9	2.8
		NO_3^-	85.6	6.7	12.3	120.7	0.6	7.9	14.6
		SO_4^{2-}	19.9	0.8	5.5	74.1	0.2	3.9	4.6
		NH_4^+	37.0	1.4	3.8	75.3	0.7	3.8	5.2
	Dianshanhu	OC	15.9	0.4	2.8	47.1	0.7	5.1	5.5
		EC	99.2	0.7	1.5	117.2	0.7	2.0	2.7
		NO_3^-	81.9	3.4	12.5	120.0	0.5	12.3	15.7
		SO_4^{2-}	-0.4	-1.3	4.9	48.6	0.4	5.4	4.0
		NH_4^+	24.2	-0.7	4.6	68.9	0.6	6.0	5.3
	Pudong	OC	-25.4	-1.6	3.0	39.4	0.7	6.5	4.9
		EC	145.9	1.3	2.1	154.2	0.5	1.5	2.8
		NO_3^-	126.5	6.3	12.0	153.1	0.5	9.1	15.3
		SO_4^{2-}	17.3	-0.5	5.5	69.9	0.3	5.1	4.7
		NH_4^+	25.0	-0.3	3.7	70.0	0.6	5.6	5.3
	Changzhou	OC	0.1	1.3	13.5	64	0.5	17.3	18.6
		EC	-0.3	-2.1	5.6	52	0.4	7.1	5.1
		NO_3^-	-0.2	-1.6	5.3	49	0.6	7.9	6.2
		SO_4^{2-}	0.0	-0.2	3.5	0.5	0.8	4.6	4.3
		NH_4^+	-0.1	-0.5	4.0	0.5	0.8	5.6	5.0
	Taiyuan	NO_3^-	0.9	6.4	14.2	1.1	0.8	7.0	13.4
		SO_4^{2-}	-0.2	-1.0	5.9	0.6	0.6	5.3	4.2
		NH_4^+	0.1	0.5	4.0	0.6	0.9	3.9	4.4

Table S15. Evaluation of $PM_{2.5}$ components simulations using CRACMM in selected cities during April 2021.

Month	Region	$PM_{2.5}$ components	NMB	MB	RMSE	NME	IOA	OBS	SIM
Apr.	Chengdu	OC	-60.5	-6.6	8.1	61.9	0.4	10.8	4.3
		EC	45.4	0.7	1.7	87.2	0.5	1.5	2.2
		NO_3^-	37.9	2.5	8.1	83.7	0.6	6.7	9.2
		SO_4^{2-}	-10.4	-0.4	2.4	50.2	0.6	3.5	3.1
		NH_4^+	-13.6	-0.5	2.7	56.3	0.7	3.6	3.1
	Chongming	NO_3^-	6.8	0.3	6.8	102.9	0.5	3.9	4.1
		SO_4^{2-}	53.7	1.7	3.8	82.6	0.4	3.2	4.9
		NH_4^+	2.9	0.1	3.0	96.5	0.5	2.0	2.1
	Dianshanhu	NO_3^-	-25.9	-6.1	12.1	82.2	0.6	11.6	5.5
		SO_4^{2-}	-3.5	-1.3	3.6	39.4	0.7	5.5	4.2
		NH_4^+	-25.3	-2.6	4.8	67.4	0.6	5.0	2.4
	Pudong	NO_3^-	-21.9	-4.5	10.0	79.5	0.5	8.6	4.1
		SO_4^{2-}	35.4	0.2	3.4	60.8	0.7	4.7	4.9
		NH_4^+	-25.4	-2.1	4.1	59.2	0.5	4.2	2.1

Table S16. Evaluation of $PM_{2.5}$ components simulations using CRACMM in selected cities during July 2021.

Month	Region	$PM_{2.5}$ components	NMB	MB	RMSE	NME	IOA	OBS	SIM
Jul.	Chengdu	OC	-52.1	-5.6	6.5	53.3	0.3	10.8	5.2
		EC	15.5	0.3	1.4	68.1	0.6	1.6	1.9
		NO_3^-	54.1	2.2	8.9	138.8	0.4	4.1	6.3
		SO_4^{2-}	-10.3	-0.4	2.4	49.7	0.5	3.6	3.2
		NH_4^+	-19.3	-0.6	3.0	72.6	0.5	3.0	2.4
	Chongming	NO_3^-	-39.6	-0.5	2.8	102.4	0.3	1.2	0.7
		SO_4^{2-}	-2.8	-0.1	2.0	60.1	0.5	2.3	2.3
		NH_4^+	-36.7	-0.3	1.3	99.4	0.4	0.7	0.4
	Dianshanhu	NO_3^-	-38.4	-1.6	3.9	80.6	0.5	2.7	1.1
		SO_4^{2-}	-36.8	-2.0	2.8	60.3	0.5	3.6	1.6
		NH_4^+	-69.7	-1.7	2.3	87.3	0.5	2.2	0.5
	Pudong	NO_3^-	-47.0	-1.6	3.4	84.0	0.4	2.3	0.7
		SO_4^{2-}	-4.8	-1.1	2.3	58.9	0.6	3.4	2.3
		NH_4^+	-69.2	-1.4	2.0	81.9	0.5	1.8	0.4
	Taiyuan	NO_3^-	-0.2	-0.9	7.9	1.0	0.4	5.0	4.1
		SO_4^{2-}	0.0	-0.1	4.5	0.6	0.5	6.4	6.3
		NH_4^+	-0.5	-1.9	3.5	0.6	0.5	4.1	2.1

Table S17. Evaluation of $PM_{2.5}$ components simulations using CRACMM in selected cities during October 2021.

Month	Region	$PM_{2.5}$ components	NMB	MB	RMSE	NME	IOA	OBS	SIM
Oct.	Chengdu	OC	-30.1	-2.8	4.5	39.8	0.5	9.4	6.6
		EC	59.9	1.3	2.5	89.7	0.3	2.2	3.5
		NO_3^-	127.9	8.3	12.7	138.5	0.5	6.5	14.9
		SO_4^{2-}	51.4	1.5	3.2	72.9	0.5	3.0	4.5
		NH_4^+	61.1	1.9	3.3	79.7	0.7	3.0	4.9
	Chongming	NO_3^-	-14.7	-0.3	3.3	77.7	0.8	1.7	1.5
		SO_4^{2-}	23.1	0.6	1.7	48.7	0.6	2.5	3.0
		NH_4^+	-41.4	-0.6	1.5	63.7	0.8	1.3	0.8
	Dianshanhu	NO_3^-	-36.5	-2.2	6.2	85.9	0.8	4.7	2.5
		SO_4^{2-}	34.8	0.1	1.6	54.0	0.8	2.8	2.9
		NH_4^+	-19.1	-0.9	2.5	85.7	0.7	2.0	1.1
	Pudong	NO_3^-	-66.3	-2.0	4.3	73.3	0.8	3.5	1.5
		SO_4^{2-}	3.5	-0.4	1.7	41.4	0.7	3.4	3.0
		NH_4^+	-65.9	-1.2	1.9	70.3	0.8	2.0	0.8
	Taiyuan	NO_3^-	0.6	4.9	10.7	0.9	0.8	8.2	13.2
		SO_4^{2-}	0.4	2.0	5.2	0.8	0.5	4.8	6.8
		NH_4^+	0.0	-0.2	3.6	0.5	0.7	4.7	4.5

Table S18. Averaged $PM_{2.5}$ evaluation metrics and the number of monitoring sites in five regions of China in January, 2021.

January	R	IOA	NMB	NME	No.
BTH	0.79	0.87	0%	38%	73
FWP	0.78	0.78	-36%	42%	50
PRD	0.35	0.45	-43%	46%	44
SCB	0.39	0.55	15%	49%	98
YRD	0.46	0.65	-1%	36%	199
Recommend Benchmark(Huang et al., 2021)	>0.60	>0.70	<±45%	<±55%	/

Table S19. Averaged $PM_{2.5}$ evaluation metrics and the number of monitoring sites in five regions of China in April, 2021.

April	R	IOA	NMB	NME	No.
BTH	0.42	0.59	-33%	42%	74
FWP	0.49	0.58	-29%	41%	52
PRD	0.57	0.55	-43%	45%	45
SCB	0.37	0.53	-5%	43%	102
YRD	0.38	0.57	-16%	37%	202
Recommend Benchmark(Huang et al., 2021)	>0.60	>0.70	<±45%	<±55%	/

Table S20. Averaged $PM_{2.5}$ evaluation metrics and the number of monitoring sites in five regions of China in July, 2021.

July	R	IOA	NMB	NME	No.
BTH	0.38	0.49	-12%	43%	72
FWP	0.24	0.47	-27%	42%	51
PRD	0.67	0.55	-50%	53%	41
SCB	0.21	0.39	13%	60%	99
YRD	0.51	0.59	-38%	50%	175
Recommend Benchmark(Huang et al., 2021)	>0.60	>0.70	<±45%	<±55%	/

Table S21. Averaged $PM_{2.5}$ evaluation metrics and the number of monitoring sites in five regions of China in October, 2021.

October	R	IOA	NMB	NME	No.
BTH	0.81	0.87	11%	39%	72
FWP	0.71	0.74	9%	48%	52
PRD	0.7	0.66	-37%	44%	44
SCB	0.61	0.57	50%	73%	99
YRD	0.74	0.81	-13%	37%	200
Recommend Benchmark(Huang et al., 2021)	>0.60	>0.70	<±45%	<±55%	/

Table S22. List of emission reductions relative to the base simulations in CB6r3_ae7 and Saprc07tic_ae7i.

Chemical Mechanism	Emission Reduction
CB6r3_ae7	Biogenic-ROC emissions set to zero
CB6r3_ae7	Benzene-, toluene-, and xylene-like emissions set to zero
Saprc07tic_ae7i	Biogenic-ROC emissions set to zero
Saprc07tic_ae7i	Benzene-, toluene-, and xylene-like emissions set to zero

References:

Huang, L., Zhu, Y., Zhai, H., Xue, S., Zhu, T., Shao, Y., Liu, Z., Emery, C., Yarwood, G., Wang, Y., Fu, J., Zhang, K., and Li, L.: Recommendations on benchmarks for numerical air quality model applications in China – Part 1: $PM_{2.5}$ and chemical species, Atmospheric Chemistry and Physics, 21, 2725-2743,doi: 10.5194/acp-21-2725-2021, 2021.

Pye, H. O. T., Place, B. K., Murphy, B. N., Seltzer, K. M., D'Ambro, E. L., Allen, C., Piletic, I. R., Farrell, S., Schwantes, R. H., Coggon, M. M., Saunders, E., Xu, L., Sarwar, G., Hutzell, W. T., Foley, K. M., Pouliot, G., Bash, J., and Stockwell, W. R.: Linking gas, particulate, and toxic endpoints to air emissions in the Community Regional Atmospheric Chemistry Multiphase Mechanism (CRACMM), Atmospheric Chemistry and Physics, 23, 5043-5099,doi: 10.5194/acp-23-5043-2023, 2023.
